# The RANI Project: A socio-normative intervention to reduce anemia in Odisha, India: A formative research protocol

**DOI:** 10.12688/gatesopenres.12808.2

**Published:** 2018-05-10

**Authors:** Erica Sedlander, Rajiv N Rimal, Sameera A. Talegawkar, Hagere Yilma, Wolfgang Munar

**Affiliations:** 1 Department of Prevention and Community Health, The George Washington University, Milken Institute School of Public Health, 950 New Hampshire Ave, Washington D.C., USA; 2 Department of Exercise and Nutrition Sciences, The George Washington University, Milken Institute School of Public Health, 950 New Hampshire Ave, Washington D.C., USA; 3 Department of Global Health, The George Washington University, Milken Institute School of Public Health, 950 New Hampshire Ave, Washington D.C., USA

**Keywords:** behavioral intervention, maternal anemia, folic acid, IFA supplements, study protocol, social norms, formative research, qualitative study

## Abstract

**Background: **More than half of women of reproductive age in India are anemic. Anemia is associated with increased risk of preterm delivery, higher maternal mortality and contributes to fatigue, which affects women’s work productivity. The World Health Organization (WHO) recommends daily oral iron and folic acid (IFA) supplements during pregnancy and weekly supplements for women of reproductive age. Government programs and global donors have distributed and promoted IFA supplements in India for over four decades. However, initial intake and compliance remain inadequate.

**Objectives: **This protocol describes the formative research phase of a larger study, called the Reduction in Anemia through Normative Innovations (RANI) Project, which will test, through a randomized controlled trial, the hypothesis that a social norms-based behavioral intervention in Odisha, India will improve uptake of IFA supplements and reduce anemia among reproductive age women as compared to usual care. The focus of this paper is on the formative research required to develop a sound intervention. We will examine socio-normative barriers to and facilitators of IFA supplement uptake.

**Methods and analysis: **Based on the Theory of Normative Social Behavior, we will adopt a mixed-method, multilevel approach. We will collect data using focus groups, in-depth interviews, observations, Rapid Participatory Ethnographic Evaluation and Research (PEER) techniques, and perceptual mapping methods. Our sample includes reproductive age women (pregnant and not pregnant), their husbands, their mothers/in law and key stakeholders. Before collecting the data, and after analyzing the results, we will hold convenings in India to engage key stakeholders in collaborative design. Following the intervention design, we will test components of the intervention, gather user feedback and fine-tune as necessary.

**Impact: **This study will contribute to the social norms and behavioral intervention research and inform policymakers about the value of adopting a socio-normative approach.

## Introduction

Anemia is a condition where the number of red blood cells and their oxygen carrying capacity is insufficient to meet the body’s physiological needs ^1^. Iron deficiency is the most significant contributor to the onset of anemia ^2^. Globally, anemia affects 1.62 billion people, which corresponds to almost 25% of the population. While young children and pregnant women have the highest rates of anemia, non-pregnant women make up the greatest number of individuals with anemia ^3^.

Anemia can lead to poor physical capacity and performance, influencing work productivity as well as increasing risk of preterm delivery and higher maternal mortality ^2, 4^. Maternal anemia could also lead to infantile iron deficiency, negatively affecting children’s physical and cognitive development ^5, 6^. Iron-deficiency anemia among women of reproductive age (WRA) leads to a substantial double burden on the potential economic development and well-being of the population. Near-term consequences of anemia among WRA include reduced labor productivity and non-workplace activity (e.g. child care, household maintenance, leisure-time physical activities) due to fatigue ^7^. In the longer term, iron deficiency during pregnancy and early childhood causes permanent reductions in children’s cognitive capacity and socio-emotional functioning that will impact their productive capacity across the life course ^8, 9^.

Anemia is a significant public health problem in India ^10^, with more than half of its women between 15 and 49 years diagnosed with the condition ^11^. Reasons for these are multifactorial and include consumption of a predominantly plant based diet ^12^; micronutrient deficiencies, such as vitamin B12 ^13, 14^; and hookworm and malarial infections ^15, 16^. Physical and cognitive productivity losses associated with anemia in India are estimated to be 6% of Gross Domestic Product (GDP ) ^17^. 

In 2012, the World Health Assembly Resolution 65.6 endorsed a Comprehensive Implementation Plan on Maternal, Infant and Young Child Nutrition (CIP), with six Global Nutrition Targets for 2025, with its second target aiming for a 50% reduction of anemia in WRA (15–49 years) ^18^. Taking iron supplements prevents anemia, and in low-income, predominantly vegetarian countries where anemia rates can be severe, it is more efficacious than increasing consumption of iron rich foods ^19^. The WHO recommends daily oral iron and folic acid (IFA) supplements during pregnancy and weekly supplements for women of reproductive age ^20^.

### Current government led programs to reduce anemia in India

India has implemented several programs to tackle anemia, the first of which is the National Nutritional Anemia Control Program, implemented in 1970, to promote regular consumption of iron rich foods and to provide iron and folic acid (IFA) supplements to pregnant and breastfeeding women ^21^. Since that time, although many nutritional programs exist in India, only few focus on anemia control.

The emergence of two programs followed in 2000, one is the Weekly Iron and Folic Acid Supplementation (WIFS) program, which provides weekly IFA supplements to adolescents through The Adolescent Girls Anemia Control Program ^22, 23^. Following this, the 12 by 12 initiative was launched in 2007 with the goal of making sure every child in India achieves a healthy hemoglobin level within 12 years ^23^. 

The Ministry of Health and Family Welfare also developed the National Iron+ Initiative Program (NIPI) to address supplementation interventions for pregnant and lactating women and supplementary nutrition for pregnant, lactating women and adolescent girls to increase protein intake ^24^. In addition to the existing programs, several researchers have designed and conducted randomized controlled trials in India to reduce anemia in WRA ^25– 28^.

Despite universal distribution and receipt, the intake of the IFA supplement remains low. Data from the 2015–2016 cycle of the India Demographic Health Data show that 91 percent of pregnant mothers reported that they received IFA supplements during their last pregnancy but only 37 percent consumed them for more than 100 days during pregnancy. Clearly, there are significant demand side barriers that contribute to low uptake and compliance.

### Barriers and facilitators to IFA uptake and compliance

Several studies, including recent investigations in Kenya and Nepal have indicated that *knowledge barriers* exist, with women reporting that they did not receive adequate information from their providers when they distributed their IFA tablets ^29, 30^. A summary of four qualitative studies in India showed that in each study, many women did not know the importance of consuming iron supplements or the link between anemia and iron supplements ^31^. Another recent study in India, reported that pregnant women were unaware of the association between anemia and maternal outcomes ^32^.

Perceived and real *side effects* have also been implicated by numerous studies as being critical barriers. Specifically, adolescents and WRA reported that side effects include gastric problems, stomach pain, weight gain and nausea ^33, 34^. Additionally, pregnant women in India report beliefs that taking too much iron may cause too much blood or a large baby, making labor more challenging ^32, 35^.

The WHO has expressed concern that the current *dose of 100mg IFA supplement* for women in India is higher than their recommendations and may therefore itself be a barrier to use ^10^. And despite the demonstrated acceptability of weekly or twice weekly supplementation in smaller trials and state-level pilot programs in India, the 2012 national scale-up of weekly IFA supplementation with 100mg iron targeting school-age youth has met resistance due to concerns regarding the gastrointestinal side effects ^36^.

Additional barriers in reducing anemia include food insecurity, which may result in a *poor-quality diet*. A 2002 qualitative study conducted in developing countries reported that dietary restrictions could vary across gender as a woman reported, “eating last” or eating “whatever is leftover” as reasons for inadequate diet ^37^.

WRA do not make decisions about their health in isolation; *social networks* can influence behaviors and family members play a crucial role in supporting the use of IFA tablets in women ^38^. Pregnant women whose spouses actively participated in antenatal visits were more likely to demonstrate a significantly higher adherence to IFA supplements than women whose husbands were not active throughout antenatal visits ^39^. Mothers and mothers-in-law can also influence the intake and adherence of IFA in pregnant women, even more than their partners as demonstrated by a formative research study among pregnant women in Bangladesh. However, when it came to money matters, husbands were involved and considered the most important decision-makers ^40^. Financial decision-making and control within a family may also have implications on a woman’s ability to obtain IFA tablets.

A growing body of literature points to social norms as a critical barrier to IFA uptake ^41, 42^. Social norms are mores or rules of behavior that are considered acceptable in a group or society ^43^. Evidence from a meta-synthesis of qualitative research on the social determinants of iron supplementation among WRA conducted in 17 countries identified social norms as one of the primary factors limiting uptake of iron supplements, particularly during pregnancy ^10^. However, to our knowledge, no studies have used a social norms approach to reduce anemia ^36^.

Social norms are different from laws in that they are negotiated through social interactions, whereas laws are codified. Traditions, like social norms are also socially negotiated, but they are more stable. The primary difference is that norms are more dynamic, and shaped and understood through communication processes ^44^ and can thereby be shaped to promote positive healthy behaviors ^45^.

Lastly, the policy context can also act as an enabler or as an obstacle for the spread of evidence-informed IFA policy innovations. The adoption of new evidence and its consideration by domestic policy actors and stakeholders remains a complex challenge for the translation of empirical evidence into policy implementation. The evidence-to-action gap is “stickier” in cases of multi- and trans-disciplinary research programs and in research programs, such as this, that deal with complex, inter-sectorial issues ^46, 47^. These challenges have been studied from various disciplinary perspectives including diffusion of innovation theory ^48, 49^ comparative policy and political science studies ^50, 51^, knowledge utilization theory and evaluation influence studies ^52– 54^, and the science of team science ^52^, among others. What has received less attention, and what comprises a significant focus of this project, are the socio-normative factors that guide policymaking. Two major knowledge gaps are of particular relevance to this study. The first is the need to better characterize the channels and mechanisms through which potential changes in population-level social norms around anemia and IFA use percolate upwards to redesign existing national and state-level policies. The second is the challenge of facilitating the adoption and utilization of the research evidence by policy actors and stakeholders in Odisha leading to system learning and improved performance in IFA policy implementation.

## Conceptual framework

Given the presence of multilevel demand side barriers to IFA use, including social norms themselves, the Theory of Normative Social Behavior (TNSB) ^55^ will underpin the formative research and subsequent intervention. The TNSB can help elucidate *when*, *how and which* norms affect health behaviors ^46^. Two key features of TNSB are particularly important. One is the distinction *between descriptive norms* and *injunctive norms*. Descriptive norms refer to individuals’ beliefs about what other people do and how often they do them. Injunctive norms are what individuals believe that others expect them to do. Descriptive norms are thought to influence behavior because of people’s desire to do the right thing or the thing that they believe most people are doing ^56^. Injunctive norms are thought to influence behavior because of individual’s motivations for connection with others ^57^. A second key feature of the TNSB is the distinction between *collective norms* (defined as a property, characteristic or behavior within a group of people) and *perceived norms* (defined as individual beliefs about other people’s actions and expectations). A key difference between the two is that collective norms operate at the societal or social network level whereas perceived norms operate at the individual level. While collective norms, which can be thought of as aggregated individual behaviors in a group, may influence perceived norms, there may be a discrepancy between what people in the group actually do and what an individual perceives that they do. For example, women of reproductive age in Odisha may believe that most women do not take IFA tablets when, in practice, many women do. Changing perceived norms around women taking IFA tablets to align with collective norms is one potential approach.

According to the TNSB, it is important to assess the source of normative influence, whether it is one’s peers, family members, or other influential people, because the closer the social distance between oneself and the referent others, the stronger the influence ^46^. This study points to the influence that mothers/mothers-in-law and husbands have on women’s likelihood to take IFA supplements. Finally, the TNSB states that the impact of norms has to be evaluated in the context of people’s aspirations to emulate others around them, the level of similarity they feel with them, the extent to which they believe they will receive benefits by engaging in the behavior, and whether they believe social sanctions will be imposed if they fail to comply. This has important implications for our formative assessment, as it points to the importance of understanding how WRA and their social network members think about IFA in terms of the costs and benefits, pressures to conform, and possible linkages between IFA or anemia and their future aspirations.

The overall effort is named the RANI Project, which stands for Reduction in Anemia through Normative Innovations.

### Research methods and analysis

***Aims and objectives***. By the end of the formative assessment period, we will have designed, adaptively tested, and refined an intervention that can be feasibly and effectively implemented to reduce iron-deficiency anemia among women of reproductive age and pregnant women in Odisha, India. To achieve this goal, the formative assessment has the following aims:

1. At the community level, to identify key contextual, administrative, policy, and service environments that either facilitate or hinder access to and use of IFA supplements among women of reproductive age and pregnant women
2. At the interpersonal and individual levels, to identify norms, referent groups, and the extent to which they act as barriers or facilitators to IFA use and compliance among reproductive age women and pregnant women
3. To design, pilot test, and subsequently modify and finalize an intervention to increase IFA use and compliance and to reduce anemia among reproductive age women (with a focus on transforming harmful norms and promoting beneficial norms)

The formative assessment will be conducted from March to December, 2018. After analyzing results, we will hold a convening workshop in July, where we will formulate a first draft of the intervention plan. We will pilot test the various components of the intervention to determine its feasibility and effectiveness. Findings from these pilot evaluations will inform and refine the intervention, which we will finalize by December, 2018.

**Figure 1.**
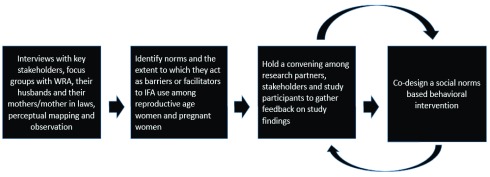
Formative Research Workflow.

Figure 1 depicts the formative research workflow.

## Research setting

### Odisha, India

**Figure 2.**
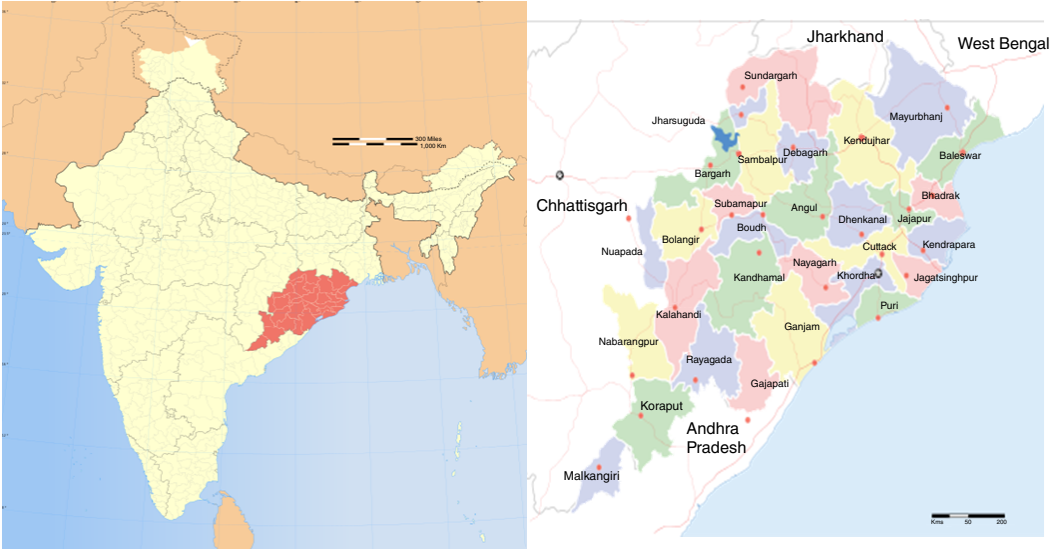
Geographic Location of Study.

Odisha is an eastern Indian state on the Bay of Bengal ( Figure 2). According to the 2015–2016 India Demographic Health Survey, 83% of households in Odisha reside in rural areas and 67% of women and 84% of men are literate. The vast majority of household heads are Hindu (95%) and 23% of households belong to a specific tribal culture. The total fertility rate (TFR) in Odisha is 2.1 children per woman. In the last five years, 60% of pregnant women had four or more antenatal care visits and 82% of women delivered in a health facility. About half (51%) of women in Odisha have anemia with a higher rate for women from a tribal culture, those following the Christian faith, and those with no schooling ^58^.

### Research team

We will conduct the research as a close partnership between the George Washington University (GW), IPE Global, our implementing partners based in Delhi and DCOR Consulting, a research firm based in Odisha, India. The research team consists of university-based researchers with expertise in qualitative research, social norms and global behavior change interventions. IPE Global and DCOR Consulting also has expertise in nutrition and SHG focused interventions in Odisha. These partnerships will be instrumental to gain access to the community and to understand the local context.

## Research design

Qualitative inquiry can improve the description and explanation of complex, real-world phenomena related to attitude and behavior change. Quantitative research alone is often insufficient to understand these complicated processes ^59, 60^. Our research team proposes to examine *why* IFA supplement use is low despite recommendations from the government and existing programs promoting its uptake and *which* existing social norms promote or hamper uptake. Additionally, surveys and quantitative methods have been the primary tool to test theories on social norms and to understand the prevalence of existing norms in communities ^61, 62^. Some theorists have suggested that more qualitative research would be useful in illuminating the process of social norm change in different situations ^63^. We respond to that call.

### Data collection modalities

We will collect qualitative data via in-depth interviews, focus groups, Participatory Ethnographic Evaluation and Research (PEER) interviews, and structured observations.

***In-depth interviews and focus groups.*** We will conduct in-depth interviews with key informants including Self-help group (SHG) leaders, health providers, teachers and health officials. We will conduct focus groups with women of reproductive age (including pregnant women), spouses, mothers, and mothers-in-law. Prior to focus group interviews participants will complete a demographic questionnaire including questions about caste. We plan to conduct approximately sixteen focus groups and twenty-four key informant interviews but the final sample size will be determined based on theoretical saturation ^64^. Table 1 shows the expected number of interviews and focus groups.

**Table 1.** Expected Number of interviews and focus groups. Preliminary Schedule of Focus Group Discussions (FGDs) and In-depth Interviews (IDIs).

Participants	Session type	# Sessions	# Participants
Self-help group leaders	IDI	4	1
Government/ministry representatives	IDI	4	1
Antenatal service providers	IDI	4	1
Secondary school teachers	IDI	4	1
Traditional Healers	IDI	4	1
Anganwadi/ASHA workers	IDI	4	1
Adolescents of reproductive age (15 – 19) years old	FGD	4	6–10
Women of reproductive age (20 – 35) years old	FGD	4	6–10
Married men (18 – 42) years old	FGD	4	6–10
Mothers-in-law	FGD	4	6–10

***Observational Data.*** Observation can allow the researcher to draw inferences about a phenomenon that they cannot obtain from direct conversation via interviews or focus groups. We will collect observational data from four venues: antenatal care (ANC) clinics, medicine stores/pharmacies, self-help groups, and outdoor food markets. Data collectors will take notes on a standard observation form (See Supplementary File 3) at one of each of the following four venues in each of the four villages (16 observations total):

Medicine store/pharmacy observations will note how well IFA tablets are stocked, where they are stocked within the store, the extent to which they are purchased, their price, and their packaging.

ANC clinic observations will note whether there are existing communications (e.g. health education posters) about anemia and/or IFA supplementation. They will also record notes on the environment including (cleanliness, crowding, wait-time, etc.). Additionally, they will observe the pharmacy within the ANC clinic and note how many IFA tablets are available, cost, packaging and stocking/stock outs. We will not inform clinic staff about the observation prior to arrival to ensure that we observe the facility in its authentic state.

Data collectors will observe SHGs and take notes about the nature of their interactions with women in the community. They will take note of how well they function, what they discuss; the kind of activities that they undertake and any challenges that they face.

Data collectors will observe outdoor food markets, as diet is an important factor to consider while studying iron-deficiency anemia. We will take photos of all food items in the market; list them out and inquire about the cost of each item.

***Perceptual Mapping.*** We will conduct perceptual mapping exercises to obtain a visual model that depicts how women and their influencers (their spouses/partners, their mothers-in-law, and others in their social network) think about IFA supplements, anemia, and other related factors, including physical and mental fatigue, and diet (see Supplementary File 4). Perceptual mapping is a technique used to elicit a mental picture held in common by members of a group. Perceptual mapping helps us understand how people construe various objects, both in terms of what meaning they give to them and how they construe the objects in relation to one another ^65, 66^. Perceptual mapping is done in three steps – attribute elicitation, scoring, and mapping.

*Step 1: Attribute Elicitation.* The purpose of this step is to extract the meaningful attributes with which people construe specific objects. For example, if people are asked to list a significant attribute pertaining to “IFA tablets,” some may point to “awful taste” as the primary attribute. For others, IFA tables may represent the idea of “medication for a better tomorrow” or “healthy” as the primary attribute. Knowing these key attributes will help us craft meaningful messages during the intervention phase. For example, we may be tempted to develop messages about IFA uptake on the assumption that it represents “no anemia” in the minds of participants. It may well be, however, that the primary attribute pertaining to taking IFA supplements for certain women may center around issues of forgetfulness or something whose impact is not easily visible. If this is the case, then our messages about IFA supplementation would be far less effective than those that tackle the issue from the perspective of linking it with more immediate positive outcomes.

For attribute elicitation, we will provide two images (on two cards), one being a reference object, of particular objects and ask people to tell us how similar or different the two are from each other (on any dimension that the participant uses). Subsequently, the reference object will be kept the same, but another second object will be shown and the same comparison will be solicited. This process will be done for all permutations of all object pairs. We anticipate having 12 objects, for a total of 55 comparisons. The objects we have currently include: IFA pill; clinic; medicine store/pharmacy; SHG; traditional healer; physicians; fatigue; prenatal care; green vegetables; meat; money; and nausea/diarrhea.

*Step 2: Scoring.* A handful of attributes are thus identified for each primary behavior of interest. Respondents are then asked to rate the importance of each attribute for each behavior or object.

*Step 3: Mapping.* Step 2 results in a *p* (number of attributes) x *q* (number of behaviors or objects) matrix that is then used to model the relative distances between behaviors or objects on a *p*-dimensional plane, thus showing the relative distances among behaviors or objects. Two behaviors or objects close to each other on a particular attribute signify their conceptual proximity. This mapping provides an understanding about how the primary behaviors or objects of interest are understood by the audience; it also specifies the relative distances among objects.

Three perceptual mapping exercises will be conducted: one among women of reproductive age (including pregnant women), one among men (who constitute the support group for the women), and one among mothers/mothers-in-law and sisters or sisters-in-law. Approximately 30 individuals per group will participate in the mapping exercise, for a total of 90 individuals.

***Rapid PEER.*** We will also conduct Rapid Participatory Ethnographic Evaluation and Research interviews (Rapid PEER), which will enable us to gain local insights into the beliefs and behaviors of beneficiaries, in the full context of their lived experience. This unique ‘insider perspective’ will ensure intervention is truly designed with the user in mind. We will train ‘ordinary’ members of the target group, in this case pregnant women and women of reproductive age, their spouses, and their mothers/mothers-in-law, after which they will serve as Peer Interviewers ^67, 68^.

To execute the Rapid PEER, we will train Peer Interviewers to carry out in-depth conversational interviews designed to obtain targeted information from others in their own social group. We will decide on five key questions that form the structure of the conversational interview process. The Peer Interviewers themselves will suggest how to form the questions from formal Odia language into a more conversational format. Non-literate Peer Interviewers will draw pictures to represent each of the five questions. We will ask the same questions at each site to at least two interviewees from the PEER interviewers social group. The informal wording and pictures to remember and ask the question may vary to suit the preference of the Peer Interviewers.

Data collection will be carried out over a one-day period, wherein the PEER interviewers will hold conversations with two same-sex friends who fit the inclusion criteria (pregnant women, women in the reproductive age group, their spouses or mothers/mothers-in-law, all living in the community, 15 years of age or older who speak Odia). Within 24 hours, researchers will debrief the PEER interviewers to obtain detailed in-depth information from each PEER interviewer on what they discussed during the interview. During the debriefing process, the research team will probe PEER interviewers for broader contextual information regarding the responses during the interview. A final workshop with PEER interviewers will explore their experiences of assisting with the formative research.

The Rapid PEER process will take 4 days at each site (two villages total):

**Table:** 

Day 1	• Rapid PEER training workshop with Peer Interviewers
Day 2	• Peer Interviewers interview their friends
Day 3	• Peer interviewers interview their friends; Rapid PEER specialist de-briefs Peer Interviewers
Day 4	• Rapid PEER specialist debriefs remaining Peer Interviewers • Final workshop with Peer Interviewers
Following days	• Rapid PEER specialist undertakes desk-based data coding and analysis

## Evidence-informed policy dialogue

We will identify key state-level stakeholders during the formative research stage. We will then design a policy dialogue and stakeholder engagement process to inform future implementation during the subsequent two years. These activities will help us collaboratively design the intervention and create a communication space for exchanging program insights and results. This process ensures that the research itself not only adds value to existing initiatives, but that research decisions are widely discussed. It further increases a-priori program ownership and the odds that stakeholders will adopt results locally. Multi-stakeholder engagement processes, are based on empirical evidence generated by studies of the diffusion of innovations, comparative policy and political science studies, knowledge utilization and evaluation studies and the science of team science ^49– 55^. During the formative stage, the aim is to characterize the factors that affect how policymaking and normative change can mutually reinforce each other ^56^.

## Data collection

### Training

We will hold an in-person training in Bhubaneswar, Odisha. Researchers from the George Washington University will conduct the training alongside research staff from DCOR Consulting. Following the training, we will pilot test all instruments and research methods over the course of two days in Gandilo Village, Khurda District located about 45 minutes outside of Bhubaneswar. We will translate and review select KII and FGDs to ensure that the participants understand the research questions and interviewers are using the skills discussed during training. After all instruments are revised, trained researchers from DCOR Consulting will collect all data in person.

We will match interviewer/moderator by gender whenever possible to build interviewer rapport ^69^. We will conduct all interviews in Odia, the local language in locations selected by our local research partners in collaboration with local officials. Each interview and focus group will be audio recorded. DCOR consulting will transcribe the interviews and focus groups from Odia to English.

### Instrument development

We designed the data collection instruments ( Supplementary File 1 – Supplementary File 4) based on the TNSB, a review of the literature on barriers and facilitators to IFA use, and feedback from the expert panel on our team. To explore IFA norms in a less personal and threatening way and to reduce social desirability bias within the focus groups, we will use vignettes, short stories about hypothetical characters who live in a rural village in Odisha. Vignettes are a simple way to explore social norms relating to a behavior ^70^. During the pilot study, we will test and revise all of the instruments as needed to ensure that the questions flow well, are culturally relevant, and capture the relevant constructs. The pilot study will also help to identify regional lexicon, which will inform revisions of the formative data collection instruments.

### Site selection

Within the state of Odisha, we plan to work in Angul district. We chose Angul for several reasons: 1) it is three hours from Bhubaneswar, the capital of Odisha, representing a rural but not isolated area 2) There are many existing SHGs 3) IFA supplements are available (as this is primarily a behavioral/demand side study); and 4) malaria and anemia rates are average compared to the rest of India. Within the district of Angul, we will conduct the formative research in the Athmalik block including four villages, two tribal and two non-tribal, Barham, Bardabar, Dudum and Tapadhol. We will also conduct the randomized control trial and intervention in Angul including but not limited to the villages from the formative research.

### Sample selection

Inclusion criteria are that participants must reside in the selected village because many of the questions pertain to norms in their communities. They must also speak either Odia or English, and they must be at least 15 years old.

### Sampling

The FGD sample will include WRA (both pregnant and not pregnant), husbands, and mothers-in-law. We plan to use a random sampling procedure to select participants for FGDs. Local Anganwadi health workers have a household enumeration of everyone who lives in each village. Based on the number of individuals needed for FGDs, against a sampling frame that will consist of the entire village, we will use a proportional skip pattern that begins with a randomly selected initial participant in order to identify households from which to select every succeeding participant for each category (e.g., mothers in law, husbands and WRA).

For the perceptual mapping exercise, we will use the same random sampling method (at the village level) and participants are only eligible to participate in one research method.

Among the key informant interviews, SHG leaders, Odisha Health Officials, teachers, and health providers, we plan to specifically employ critical case or “reputational” sampling where cases will be chosen based on specialized knowledge or expertise. These interviews will be conducted with individuals who have expertise with respect to a specific perspective on this issue ^71, 72^. Therefore, we will conduct key informant interviews with SHG leaders, ANC clinic providers who serve pregnant and reproductive age women, health officials who work on anemia or IFA distribution/education in Odisha and front line IFA dispensary workers. This sampling strategy will ensure that we interview the most knowledgeable key informants. Our sampling strategy will be refined in consultation with our local partners and Anganwadi health workers, who will recommend key informants who meet these criteria.

We will conduct Rapid PEER interviews in two villages within the selected district. In each site we will purposively choose six PEER interviewers (one each from women of reproductive age (15 – 25 years old), women of reproductive age (26 – 49 years old), pregnant women (15 – 49 years old), younger married men (15 – 25 years old), older married men (26–49 years old) and mothers-in-law). Each interviewer will interview or hold conversations with two interviewees (with same-sex friends who fit the previously discussed inclusion criteria). Within the two villages, we will train 12 PEER interviewers who will conduct 24-interviews.

## Analysis

We will use an iterative approach to data collection and data analysis, whereby analysis and data collection will happen concurrently. This will allow us to determine when thematic saturation occurs and no new themes are emerging from the data ^56^. Initially, we will read transcripts and create memos, written logs used to capture reflection and analytic insights (Groenewald, 2008) ^73^. Findings from all data collection sources, semi-structured interviews, Rapid PEER interviews, focus groups, observation, and perceptual mapping, will be triangulated to examine whether or not these different methods support the same identified themes. Using different data sources will also allow us to gain information about different aspects of the phenomenon ^74^.

Following the procedures outlined by Guest *et al.*, 2011 ^75^, we will conduct applied thematic analysis to characterize the knowledge, attitudes and behaviors relevant to IFA use. We chose applied thematic analysis because our overall study aim, to inform an anemia reduction intervention, is applied in nature. Experienced qualitative researchers from both The George Washington University and DCOR Consulting will independently review transcripts to develop an initial codebook and modify the codebook as themes emerge. We will use both inductive and deductive coding to categorize concepts. Specific a priori codes will be used to identify text related to our research questions and additional codes will be added to the codebook based on new themes that emerge during coding. Using NVivo v.11, we will identify themes by comparing codes and content across sources, and by running specific word queries, associations between themes, and creating hierarchal visual displays of codes to identify linkages and patterns in the data.

We will analyze perceptual mapping data with multidimensional scaling techniques, which seek commonalities in responses that can be clustered together into groups, much like factor analysis, that have low intra-group and high intergroup variances. First, we will map clusters on a multiplane axis and iteratively identify the underlying dimension. Next, we will compare emerging dimensions with themes emerging from the qualitative analysis. Lastly, we will discuss and identify the underlying dimension with the highest validity and intervention utility.

Finally, we will use matrix analysis as a tool to display and further develop our results. Identified themes will be organized using descriptive matrix analyses that visually display the range of related responses ^76^. This will allow for a comprehensive analysis of the data and ensure that we not only focus on the majority responses among participants, but also the outliers.

We will analyze the Rapid PEER interviews following these steps:

- To keep the conversation flowing, the Peer Interviewers will not take notes during the interviews but simply remember the conversation. Shortly after the interviews (the same day or the next day), the facilitator from the study team will meet and ask the Peer Interviewers about what their friends said about the topic. The facilitator will take detailed notes during these de-briefing sessions.
- The notes (originally written in Odia) will be translated verbatim in English. After translation, the research team will read all of the notes and identify the primary themes that emerged from the interviews. Next, they will assign themes to text segments.
- They will then identify relevant barriers and facilitators to IFA use and select illustrative quotes to include in a final report.

To test the validity of our findings and the existence of potential threats to those conclusions, we will conduct several validity checks including:

- *Long-term involvement in the community.* Our formative research is planned to continue for six months to provide adequate time to pilot test and tailor the data collection instruments, to return to follow up on initial findings, to provide adequate time to complete data collection and to reach thematic saturation.
- *Rich data.* We will use verbatim transcripts of the interviews and focus groups as well as descriptive notes for the perceptual mapping and observation.
- *Respondent validation.* We will hold a convening in Odisha to present and get feedback on our findings from participants, key stakeholders and research partners.
- *Triangulation.* We will triangulate our data by collecting data from a diverse range of individuals and use a variety of data collection methods. Additionally, our research teams have diverse backgrounds and bring multiple perspectives to the data analysis. Researchers from both GWU and DCOR consulting will co-analyze data.

Throughout the design, data collection and analysis, we will refer to the consolidated criteria for reporting qualitative research (COREQ): a 32-item checklist for interviews and focus groups to ensure study rigor and credibility ^77^. The quality of study reporting has been improved through formal reporting frameworks, like the COREQ ^78^.

**Strengths and limitations of this study**

- This study will provide evidence-based recommendations to guide the development of a social norms-based anemia reduction intervention.
- This study will explore multilevel (individual, interpersonal, community, and environment) barriers and facilitators to iron and folic acid uptake and compliance.
- We will conduct this efficacy trial in one state in India. Therefore, generalizability may be limited.

## Ethics and dissemination

The Institutional Review Board at The George Washington University and the DCOR Institutional Ethics Committee approved this study. All participants will go through a verbal and written informed consent process before data collection. We will disseminate study findings locally in Odisha, at international conferences and publish in peer-reviewed journals.

## Data availability

No data is associated with this article.

## Supplementary material

**Supplementary material:** File 1- Focus Group Guide. File 2- Key Informant Interview Guide. File 3- Structured Observation Check Lists. File 4- Perceptual Mapping Questionnaire.
