# Supplementary material for: The RANI Project: A socio-normative intervention to reduce anemia in Odisha, India: A formative research protocol
Source: Gates Open Res. 2018 May 10;2:15. Originally published 2018 Mar 20. [Version 2] doi: 10.12688/gatesopenres.12808.2 (PMC5906750; doi:10.12688/gatesopenres.12808.2)
Supplement: Supplementary file 1 [file gatesopenres-2-13894-s0000.tgz › fc760b8c-8c57-4732-9bf6-83edefcf24a1.zip!/Supplementary file 1.pdf]

Focus Group/Interview Guide

Before Beginning the Interview:
ସାକ୍ଷାତକାର ଆରମ୍ଭ ପୂର୍ବରୁ:

- Welcome participant and introduce yourself
  ଅଂଶଗ୍ରହଣକାରୀଙ୍କୁ ସ୍ଵାଗତ କରନ୍ତୁ ଏବଂ ନିଜର ପରିଚୟ ଦିଅନ୍ତୁ |

Hello, and thank you for speaking with me today. My name is ______ and I am a field researcher at DCOR. I am conducting a research study to understand the attitudes and beliefs of people in this community.
ନମସ୍କାର, ଏବଂ ଆପଣ ଆଜି ମୋ ସହିତ କଥା ହେଉଥିବାରୁ ଆପଣଙ୍କୁ ଧନ୍ୟବାଦ ଦେଉଛି | ମୋ ନାମ ହେଉଛି ____________ଏବଂ ମୁଁ DCOR ସଂସ୍ଥାର ଜଣେ କ୍ଷେତ୍ର ଅନୁସନ୍ଧାନକାରୀ ଭାବେ କାମ କରୁଅଛି | ମୁଁ ଏହି ଗ୍ରାମରେ ଲୋକମାନଙ୍କର  ମନୋଭାବ ଏବଂ ଧାରଣା ଗୁଡିକୁ ଜାଣିବା ପାଇଁ ଗୋଟିଏ ଅନୁସନ୍ଧାନ କରୁଅଛି |

Thank you so much for agreeing to participate and taking time out of your day.
ଆପଣମାନେ ଏହି ଆଲୋଚନାରେ ଭାଗ ନେବା ପାଇଁ ସହମତି ଦେଇଥିବାରୁ ଏବଂ ଆପଣ ଆଜି ସମୟ ବାହାର କରି ଆସିଥିବାରୁ ଧନ୍ୟବାଦ ଦେଉଛି |

Do you have any questions about the research and your participation before we start?
ଆମେ ଆଲୋଚନାଟି ଆରମ୍ଭ କରିବା ପୂର୍ବରୁ, ଏହି ଅନୁସନ୍ଧାନ ବିଷୟରେ ଏବଂ ଆପଣଙ୍କ ଅଂଶଗ୍ରହଣ କରିବା ଉପରେ କିଛି ପ୍ରଶ୍ନ ଅଛି କି ?

- Review key points, ethics and confidentiality policy:
  ମୁଖ୍ୟ ବିନ୍ଦୁଗୁଡିକ ଉପରେ, ଆଚାର ବିଚାର ଉପରେ ଏବଂ ଗୋପନୀୟତା ସମ୍ବନ୍ଧୀୟ ନୀତି ଗୁଡିକ ଉପରେ ଅବଲୋକନ କରନ୍ତୁ |

- You will not gain any direct benefit from participating in this research; however, we hope our results will be used to reduce anemia in Odisha.
  ଏହି ଅନୁସନ୍ଧାନରେ ଭାଗ ନେବା ଦ୍ବାରା ଆପଣଙ୍କୁ ସିଧା ସଳଖ ଭାବେ କୌଣସି ଲାଭ ମିଳିବ ନାହିଁ : କିନ୍ତୁ ଆମେ ଆଶା କରୁଛୁ, ଏହି ଅନୁସନ୍ଧାନର ଫଳାଫଳ ଓଡିଶାରେ ରକ୍ତହିନତା ଦୂର କରିବା ପାଇଁ ବ୍ୟବହାର କରାଯିବ |

- We do not anticipate you will experience any risks but please feel free to not answer any questions that you are uncomfortable with or to stop the interview at anytime.
  ଆମେ ଆଶା କରୁନାହୁଁ କି ଆପଣ ଏହି ଆଲୋଚନାରେ କିଛି ସଙ୍କଟ / ଅସୁବିଧା ଅନୁଭବ କରିବେ | ମାତ୍ର, ଯଦି କୌଣସି ପ୍ରଶ୍ନର ଉତ୍ତର ଦେବା ପାଇଁ ଆପଣ ଅସୁବିଧା ମନେ କରନ୍ତି, ତାହାହେଲେ ତାହାର ଉତ୍ତର ଦେବା ପାଇଁ ଆପଣ ବାଧ୍ୟ ନୁହଁନ୍ତି ବା ଯେକୌଣସି ସମୟରେ ଏହି ଆଲୋଚନାଟିରୁ ଓହରି ଯାଇପାରିବେ |

- The discussion in this interview is completely confidential
  ସାକ୍ଷାତକାରରେ ଯାହା ବି କଥା ହେବା ତାହାକୁ ସମ୍ପୁର୍ଣ୍ଣ ରୁପେ ଗୋପନୀୟ ରଖାଯିବ |

- Your responses will be kept confidential and your name will not be cited on any written materials coming out of this study.
  ଆପଣଙ୍କ ଉତ୍ତରଗୁଡିକୁ ଗୋପନୀୟ ରଖାଯିବ ଏବଂ ଆପଣଙ୍କର ନାମ ଏହି ଅନୁସନ୍ଧାନ ଦ୍ଵାରା ପ୍ରସ୍ତୁତ କରାଯାଇଥିବା କୌଣସି ଲିଖିତ କାଗଜପତ୍ରରେ ଉଲ୍ଲେଖ କରାଯିବ ନାହିଁ |

- There are no rights or wrong answers to the questions I am going to ask you. All experiences are important
  ମୁଁ ପଚାରୁଥିବା ପ୍ରଶ୍ନର କୌଣସି ଠିକ୍ କିମ୍ବା ଭୁଲ୍ ଉତ୍ତର ନାହିଁ | ଆପଣଙ୍କ ସମସ୍ତ ଉତ୍ତର/ଅନୁଭବ ଗୁଡିକ ଗୁରୁତ୍ୱପୂର୍ଣ୍ଣ ଅଟେ |

- The interview is being tape recorded so that we can accurately capture what you're saying, but no one besides the research team will have access to it.
  ଏହି ଆଲୋଚନାଟି ଆମେ ରେକର୍ଡିଂ କରୁଛୁ ଯାହା ଫଳରେ ଆପଣ କହିଥିବା ସମସ୍ତ କଥାକୁ ଆମେ ଲେଖିପରିବୁ | କିନ୍ତୁ ଏହି ଅନୁସନ୍ଧାନ ସହିତ ଜଡିତ ନଥିବା ବ୍ୟକ୍ତି ଏହାକୁ ବ୍ୟବହାର କରିପାରିବେ ନାହିଁ |

- You are free not to answer any questions that make you feel uncomfortable or to stop the interview at any time
  କୌଣସି ପ୍ରଶ୍ନର ଉତ୍ତର ଦେବା ପାଇଁ ଆପଣ ଅସୁବିଧା ମନେ କରନ୍ତି, ତାହାର ଉତ୍ତର ଦେବା ପାଇଁ ଆପଣ ବାଧ୍ୟ ନୁହଁନ୍ତି ବା ଯେକୌଣସି ସମୟରେ ଏହି ଆଲୋଚନାଟିରୁ ଓହରି ଯାଇପାରିବେ |

- We expect the interview to last about one hour and a half
  ଆମେ ଆଶା କରୁଛୁ  ଏହି ଆଲୋଚନାଟି ଦେଢ଼ ଘଣ୍ଟା ପର୍ଯ୍ୟନ୍ତ ସମୟ ଲାଗିବ |

## Warm-up Questions

I'm new to this community, [name District], can you tell me a little bit about it? For example, what kind of food do people eat here? And what do people do to earn money?
ମୁଁ ଆପଣଙ୍କ ସମୁଦାୟରେ/ଗ୍ରାମରେ ନୂଆ, (ଜିଲ୍ଲାର ନାମ), ଆପଣଙ୍କ ସମୁଦାୟ/ଗ୍ରାମ ବିଷୟରେ କିଛି କହିପାରିବେ କି ? ଉଦାହରଣସ୍ୱରୂପ, କେଉଁ ପ୍ରକାରର ଖାଦ୍ୟ ଏଠାରେ ଲୋକମାନେ ଖାଆନ୍ତି ? ଏବଂ ଲୋକମାନେ ରୋଜଗାର ପାଇଁ କଣସବୁ କରନ୍ତି ?

## Women's Role in the Community
## ଗ୍ରାମରେ/ସମୁଦାୟରେ ମହିଳାମାନଙ୍କର ଭୂମିକା

What are some of the typical things that women in this community do throughout the day? (probe: things like cooking, cleaning, taking care of kids, working outside the home, spending time with her husband).
ଆପଣଙ୍କ ଗ୍ରାମରେ/ସମୁଦାୟରେ ମହିଳାମାନେ ସାଧାରଣତଃ ଗୋଟିଏ ଦିନ ଭିତରେ କଣ ସବୁ କାର୍ଯ୍ୟ କରିଥାନ୍ତି ? (ପଚାରନ୍ତୁ: ଯେପରିକି ରୋଷେଇ କରିବା, ସଫା କରିବା, ପିଲାଙ୍କର ଯତ୍ନ ନେବା, ଘର ବାହାରେ କାର୍ଯ୍ୟ କରିବା, ସ୍ୱାମୀଙ୍କ ସହିତ ସମୟ ବିତାଇବା)

Do women in this community have enough energy or time to these things each day?
ପ୍ରତ୍ୟକ ଦିନ ଏହି କାର୍ଯ୍ୟ ସବୁ କରିବା ପାଇଁ, ଆପଣଙ୍କ ସମୁଦାୟରେ ମହିଳାମାନଙ୍କର ପର୍ଯ୍ୟାପ୍ତ ଶକ୍ତି ବା ସମୟ ଥାଏ କି ?

- Look after her kids?
  ଛୁଆମାନଙ୍କର ଯତ୍ନ ନେବା ?
- Work outside the home?
  ଘର ବାହାରେ କାର୍ଯ୍ୟ କରିବା ?
- Cook?
  ରୋଷେଇ କରିବା ?
- Spend time with her husband?
  ସ୍ୱାମୀଙ୍କ ସହିତ ସମୟ ବିତାଇବା ?

- Have more intimate time with her husband?
  ସ୍ୱାମୀଙ୍କ ସହିତ ଅଧିକ ଅନ୍ତରଙ୍ଗ ସମୟ ବିତାଇବା ?

How are men and women different in this community?
ଆପଣଙ୍କ ସମୁଦାୟ/ଗ୍ରାମରେ ପୁରୁଷ ଏବଂ ମହିଳାମାନେ ପରସ୍ପର ଠାରୁ କିପରି ଭିନ୍ନ ?

For women's life in general, are things getting better or worse?
ସାଧାରଣତଃ, ମହିଳାମାନଙ୍କର ଜୀବନରେ ଘଟୁଥିବା ଜିନିଷ ଗୁଡିକ ଭଲ ବା ଖରାପ ହେଉଛି କି ?

- in her role as a mother?
  'ମା' ହିସାବରେ ତାଙ୍କ ଭୂମିକା ?
- in her role as a wife?
  'ସ୍ତ୍ରୀ' ହିସାବରେ ତାଙ୍କ ଭୂମିକା ?
- in her role as a daughter in law?
  'ବୋହୂ' ହିସାବରେ ତାଙ୍କ ଭୂମିକା ?

What are some of the concerns that women in this community have? (probe: concerns about health, money, their family, themselves, their future)
ଆପଣଙ୍କ ସମୁଦାୟର ମହିଳାମାନଙ୍କର କଣ ସବୁ ଚିନ୍ତା ରହିଛି ? (ପଚାରନ୍ତୁ: ଯେପରିକି ସ୍ୱାସ୍ଥ୍ୟ, ଟଙ୍କା, ତାଙ୍କ ପରିବାର, ନିଜ ପାଇଁ, ଭବିଷ୍ୟତ ପାଇଁ ଚିନ୍ତା ସବୁ)

- Some people think that (woman's name) should first take care of her husband and kids before she worries about her own health. Other people think that she should first take care of her own health.

  କିଛି ଲୋକ ଭାବନ୍ତିକି, ଯେ (ମହିଳା ଙ୍କ ନାମ) ନିଜର ସ୍ୱାସ୍ଥ୍ୟଯତ୍ନ ପାଇଁ ଚିନ୍ତା କରିବା ପୁର୍ବରୁ ପ୍ରଥମେ ନିଜ ସ୍ୱାମୀ ଏବଂ ଛୁଆଙ୍କ ଯତ୍ନ ନେବା ଜରୁରୀ | ଅନ୍ୟକିଛି ଲୋକ ଭାବନ୍ତିକି ଯେ ସେ ପ୍ରଥମେ ନିଜ ସ୍ୱାସ୍ଥ୍ୟର ଯତ୍ନ ନେବା ଜରୁରୀ |

  How do you think most women in this community feel about that? What about most men?
  ଆପଣଙ୍କ ଗ୍ରାମରେ ମହିଳା ମାନେ ଏହି ବିଷୟରେ କଣ ଭାବନ୍ତି ? ପୁରୁଷ ମାନେ କଣ ଭାବନ୍ତି ?

  In some families in Odisha, women eat after their husband, children and mother in law eat. In others, women eat at the same time. What do you think about that? Is this changing?
  ଓଡ଼ିଶାରେ ଥିବା କିଛି ପରିବାରରେ, ମହିଳାମାନେ ତାଙ୍କ ସ୍ୱାମୀ, ଛୁଆ ଏବଂ ଶାଶୂ ଖାଇସାରିଲା ପରେ ଖାଆନ୍ତି | ଅନ୍ୟ କିଛି ପରିବାରରେ, ମହିଳାମାନେ ସେମାନଙ୍କ ସହିତ ଏକା ସମୟରେ ଖାଇଥାଆନ୍ତି | ଏହି ବିଷୟରେ ଆପଣମାନେ କଣ ଭାବନ୍ତି ? ଏହା ପରିବର୍ତ୍ତନ ହେଉଛି କି?

## Anemia and IFA Knowledge
ରକ୍ତହୀନତା ଏବଂ ଆଇରନ ବଟିକା ଉପରେ ଜ୍ଞାନ

(Show an IFA tablet and liquid IFA)
(ଗୋଟିଏ ଆଇରନ ବଟିକା ବା ସିରପ୍ ଦେଖାନ୍ତୁ)

Focus Group/Interview Guide

Can someone tell me what this is? (probe: What does it do?)
କେହି ମୋତେ ବୁଝେଇପାରିବେ କି ଏହା କଣ? (ପଚାରନ୍ତୁ: ଏହା କଣ ସାହାଯ୍ୟ କରେ ?)

Have you ever heard of anemia?
ଆପଣ ମାନେ ଆନେମିଆ ବିଷୟରେ କେବେବି ଶୁଣିଛନ୍ତି କି ?

Many people in this community may not use the word "anemia" but they may have other words or phrases to talk about anemia. Can you tell me what some of those phrases are?
ଗ୍ରାମରେ ବହୁ ଲୋକ ଆନେମିଆ ଶବ୍ଦର ବ୍ୟବହାର କରୁନଥିବେ କିନ୍ତୁ ଆପଣମାନେ ଏହା ପାଇଁ ଅନ୍ୟ କୌଣସି ଶବ୍ଦ ବା ବାକ୍ୟର ବ୍ୟବହାର କରୁଥିବେ | ଆପଣ ମାନେ ଏହାକୁ ଆପଣଙ୍କ ଭାଷାରେ କଣ କୁହନ୍ତି, କହିବେ କି ?

Tell me a little bit about what happens when someone has <u>anemia</u> (use the word or phrase identified above instead of "anemia"). Tell me how this person feels or how this person acts when they have <u>anemia</u>.
ଯଦି ଜଣକୁ ରକ୍ତହିନତା ହୋଇଥାଏ ତେବେ କଣ ହୁଏ, ସେ ବିଷୟରେ ମତେ କିଛି କହିବେ କି ? (ଆନେମିଆ ଶବ୍ଦ ବଦଳରେ ସେମାନେ କହୁଥିବା ଶବ୍ଦର ବ୍ୟବହାର କରନ୍ତୁ) | ଯେତେବେଳେ ଜଣକର ରକ୍ତହୀନତା ଥିବ, ସେ ଲୋକ କେମିତି ଅନୁଭବ କରେ ବା ସେ କେମିତି କାମ କରେ ?

What do you think causes <u>anemia</u>? (probe: Not eating enough iron rich foods?)
ରକ୍ତହିନତାର କାରଣ କଣ ହୋଇପାରେ ବୋଲି ଆପଣ ଭାବୁଛନ୍ତି ?( ପଚାରନ୍ତୁ : ଅଧିକ ମାତ୍ରାରେ ଆଇରନ୍ ଯୁକ୍ତ ଖାଦ୍ୟ ନଖାଇବା ଦ୍ବାରା ହୋଇଥାଏ)

What do you think makes anemia go away? (Probe: Can IFA tablets help? How about changing your diet? What kind of foods might help it go away? )
ଆପଣ କଣ ଭାବୁଛନ୍ତି ଯାହା ରକ୍ତହୀନତାକୁ ଦୂର କରିବାରେ ସାହାଯ୍ୟ କରିଥାଏ ? (ପଚାରନ୍ତୁ: ଏଥିପାଇଁ ଆଇରନ ବଟିକାସାହାଯ୍ୟ କରିପାରିବ କି ? ଆପଣଙ୍କ ଖାଦ୍ୟରେ ପରିବର୍ତ୍ତନ ଏହାକୁ ଦୂର କରିପାରିବ କି ? କଣସବୁ ଖାଦ୍ୟ ଏହାକୁ ଦୂର କରିବାରେ ସାହାଯ୍ୟ କରି ପାରିବ ?

## <u>Anemia Related Behavior</u>
## <u>ରକ୍ତହୀନତା ସମ୍ବନ୍ଧୀୟ ବ୍ୟବହାର</u>

Ok, now we're going to make up a character. She is a female aged 23 years old. What should we call her? (Ask for suggestions and decide on a name together). She just got married and is 3 months pregnant with her first child. Her doctor just told her she has anemia.
ହଉ, ବର୍ତ୍ତମାନ ଆମେ ଗୋଟିଏ ନାଟକୀୟ ଚରିତ୍ରଟିଏ ମନରେ ଗଢିବା | ସେ ଜଣେ ମହିଳା ଯାହାଙ୍କର ଆୟୁ ୨୩ବର୍ଷ | (ସମସ୍ତଙ୍କ ପରାମର୍ଶରେ ମହିଳାଙ୍କର ନାମଟିଏ ରଖନ୍ତୁ) | ସେ ଏବେ ବିବାହ କରିଛନ୍ତି ଏବଂ ୩ମାସ ହେଲା ଗର୍ଭବତୀ ଅଛନ୍ତି | ତାଙ୍କର ଆନେମିଏ\ରକ୍ତହୀନତା ଅଛି ବୋଲି ତାଙ୍କୁ ଡାକ୍ତର ଏବେ କହିଥିଲେ |

The doctor told her to take iron tablets.
ଡାକ୍ତର ତାଙ୍କୁ ଆଇରନ ବଟିକା ଖାଇବା ପାଇଁ କହିଥିଲେ ?

- If (same woman's name) wanted to get IFA tablets, where could she get them? How difficult is it to *get* IFA tablets?
  ଯଦି (ମହିଳାଙ୍କ ନାମ) ଆଇରନ ବଟିକା ଦରକାର ମନେ କରନ୍ତି ତାହାଲେ ସେ କେଉଁଠାରୁ ପାଇପାରିବେ? ଏହି ବଟିକା ପାଇବା ପାଇଁ କେତେ କଷ୍ଟସାଧ ହୋଇଥାଏ |

  - Can you tell me what she likes about taking the tablets and what she doesn't like? (PROBE by asking more likes and dislikes)
    ଆପଣ ମାନେ ମତେ କହିପାରିବେ କି ଆଇରନ ବଟିକା ଖାଇବାକୁ ନେଇ ସେ କଣ ପସନ୍ଦ କରନ୍ତି ଓ କଣ ପସନ୍ଦ କରନ୍ତି ନାହିଁ | ( ଅଧିକ ପସନ୍ଦ ଓ ନାପସନ୍ଦ ଗୁଡିକ ପଚାରି ଜାଣିବାକୁ ଚେଷ୍ଟା କରନ୍ତୁ)

  - Apparently, some women stop taking those tablets. Can you tell me why they stop?
    ଯେମିତିବି ହେଉ, କିଛି ମହିଳା ମାନେ ଏହି ବଟିକା ଖାଇବା ବନ୍ଦ କରି ଦିଅନ୍ତି | ଆପଣ ମାନେ କହିପାରିବେ କି ସେମାନେ କାହିଁକି ବଟିକା ଖାଇବା ବନ୍ଦ କରି ଦିଅନ୍ତି ?

  - Can you tell me what good things may happen if someone takes the tablets?
    ଆପଣମାନେ ମତେ କହି ପାରିବେ କି ଯଦି ଜଣେ ବଟିକା ଖାଏ, ତେବେ ତାର କଣ ଭଲ ହେବ ?

  - What do you think would help her take the tablets? Do you think (woman's name)'s husband will support her to take the tablets? Not support her to take them? Or not say anything? (Ask for examples)
    ଆପଣ ମାନେ ଭାବୁଛନ୍ତି କି, (ମହିଳାଙ୍କ ନାମ) ଙ୍କ ସ୍ବାମୀ ତାଙ୍କୁ ବଟିକା ଖାଇବା ପାଇଁ ଉତ୍ସାହିତ କରିବେ ? ବଟିକା ଖାଇବା ପାଇଁ ଉତ୍ସାହିତ କରିବେ ନାହିଁ ? କିମ୍ବା କିଛି କହିବେ ନାହିଁ ? (କିଛି ଉଦାହରଣ ଦେବା ପାଇଁ କୁହନ୍ତୁ)

  - What about (woman's name)'s mother in law? Will she support her in taking the tablets? Why or why not?
    ମହିଳା (ମହିଳାଙ୍କ ନାମ) ଙ୍କ ଶାଶୂ ଆଇରନ ବଟିକା ଖାଇବା ବିଷୟରେ କଣ କହିବେ ? ସେ ତାଙ୍କୁ ବଟିକା ଖାଇବା ପାଇଁ ସାହାଯ୍ୟ କରିବେ କି ? କାହିଁକି ବା କାହିଁକି ନୁହେଁ |

## IFA Norms

ଆଇରନ ବାଟିକାକୁ ନେଇ ନିୟମାବଳୀ

Is Anemia a problem in the community? If yes How much of a problem?
ଆପଣଙ୍କ ଗ୍ରାମରେ ଆନେମିଆ/**ରକ୍ତହୀନତା ଗୋଟିଏ** ସମସ୍ୟା କି **?** ଯଦି ହଁ, କେତେ ବଡ ସମସ୍ୟା **?**

- In general, who is typically expected to take IFA tablets? (Probe: pregnant women, adolescent girls, non-pregnant women?)
  ସାଧାରଣତଃ, କାହାକୁ ଆଇରନ ବଟିକା ଖାଇବା ପାଇଁ ଆଶା କରାଯାଇଥାଏ ? (ପଚାରନ୍ତୁ: ଗର୍ଭବତୀ ମହିଳା, କିଶୋରୀ ବାଳିକା, ଗର୍ଭବତୀ ନଥିବା ସ୍ତ୍ରୀ ଲୋକମାନେ)

- Please think about most women like (name) who live in this community. How many of them take IFA tablets? Some? Few? Most?
  ଏହି (ମହିଳାଙ୍କ ନାମ) ପରି, ଗ୍ରାମରେ ରହୁଥିବା ଅଧିକାଂଶ ମହିଳାଙ୍କ ବିଷୟରେ ଭାବନ୍ତୁ | ସେମାନଙ୍କ ମଧ୍ୟରୁ କେତେଜଣ ଆଇରନ ବଟିକା ଖାଆନ୍ତି ? କିଛି ଜଣ ? ଅଳ୍ପ ଜଣ ? ଅଧିକାଂଶ ଜଣ ?

- Is there anyone in (woman's name) family (or her husband's family) that she can talk to about taking IFA tablets? How much do you think she'll listen to what they have to say about IFA tablets?
  ଏମିତି କେହି (ମହିଳାଙ୍କ ନାମ)ଙ୍କ ପରିବାରରେ(ବା ତାଙ୍କସ୍ୱାମୀଙ୍କର ପରିବାରରେ) ଅଛନ୍ତି, ଯାହା ସାଙ୍ଗେ ସେଆଇରନ ବଟିକ ଖାଇବା ବିଷୟରେ କଥା ହୋଇପାରିବେ ? ଆପଣଙ୍କୁ କଣ ଲାଗୁଛି, ଯାହାତାଙ୍କ ପରିବାରର ସଦସ୍ୟମାନେ ଆଇରନ ବଟିକା ବିଷୟରେ କହୁଥିବେ,ସେ କେତେ ମାତ୍ରାରେ ଗ୍ରହଣ କରିବେ?

**<u>Information Sources</u>**
<u>ସୂଚନାର ଉତ୍ସ</u>

Imagine another woman who is not pregnant but interested in learning more about her health. What should we call her? (Ask for suggestions and decide on a name together).
ଆଉଜଣେ ମହିଳାଙ୍କ ବିଷୟରେ କଳ୍ପନା କରନ୍ତୁ ଯିଏ ଗର୍ଭବତୀ ନୁହଁନ୍ତି | ମାତ୍ର ସେ ନିଜ ସ୍ୱାସ୍ଥ୍ୟ ବିଷୟରେ ଜାଣିବା ପାଇଁ ଇଚ୍ଛୁକ | (ସମସ୍ତଙ୍କ ପରାମର୍ଶରେ ସେହି ମହିଳାଙ୍କର ଗୋଟିଏ ନାମ ଦିଅନ୍ତୁ |)

Where does she go for information about health related matters?
ସେ ସ୍ୱାସ୍ଥ୍ୟ ସମ୍ବନ୍ଧୀୟ ତଥ୍ୟସବୁ ଜାଣିବା ପାଇଁ କେଉଁଠାକୁ ଯାଆନ୍ତି ?

Where can she get information about pregnancy, anemia, and IFA tablets from?
ସେ କେଉଁଠାରୁ ଗର୍ଭାବସ୍ଥା, ରକ୍ତହୀନତା ଏବଂ ଆଇରନ ବଟିକା ବିଷୟରେ ସୂଚନା ପାଇପାରିବେ ?

- How easy is it for her to get information about pregnancy, anemia, and/or IFA tablets? What difficulties would come up when trying to get this information?
  ତାଙ୍କ ପାଇଁ ଗର୍ଭାବସ୍ଥା, ରକ୍ତହୀନତା, ଆଇରନ ବଟିକା ବିଷୟରେସୂଚନା ପାଇବା କେତେ ସହଜ ହୋଇଥାଏ ? ଏହି ସୂଚନା ସବୁ ପାଇବାକୁ ଚେଷ୍ଟା କଲାବେଳେ କଣ ସବୁ ସମସ୍ୟାର ସମ୍ମୁଖୀନ ହୋଇଥାନ୍ତି ?

- When a woman gets IFA tablet, what kind of information does she get about them?
  ଯେତେବେଳେ ଜଣେ ଆଇରନ୍ ବଟିକା ଆଣନ୍ତି, ସେ ଏହା ବିଷୟରେ କେଉଁ ପ୍ରକାରର ସୂଚନା ସେହି ସମୟରେ ପାଇଥାନ୍ତି ?

- Probe: Dose, when to take or why to take it?
  ପଚାରନ୍ତୁ: କେତେବେଳେ ଖାଇବା କଥା ବା କାହିଁକି ଖାଇବା କଥା ?

- For those of you that participate in self-help groups, can you tell me what kind of things you discuss?
  ଯେଉଁମାନେ ସ୍ୱୟଂସହାୟକ ଦଳରେ ଅଂଶଗ୍ରହଣ କରନ୍ତି, ଆପଣ କଣ ସବୁ ବିଷୟ ଉପରେ ଆଲୋଚନା କରନ୍ତି ?

## Closing

ସମାପନ

That is the end of the questions I have for you, but do you have anything else you'd like to add to the discussion? Any little stories about anemia or IFA use in this community?
ମୋର ପ୍ରଶ୍ନ ପଚାରିବା ଶେଷ ହେଲା, କିନ୍ତୁ ଆପଣମାନେ ଏହି ଆଲୋଚନାରେ ଆଉ କିଛି କହିବାକୁ ଚାହୁଁଛନ୍ତି କି ? ରକ୍ତହୀନତା କିମ୍ବା ଆଇରନ ବଟିକା ଉପରେ, ଆପଣଙ୍କ ଗ୍ରାମରେ ହୋଇଥିବା କିଛି ଘଟଣା ବା କାହାଣୀ କହିବେ କି ?

As a reminder, please do not share anything we spoke about today with anyone outside of this group.
ଆପଣଙ୍କୁ ମନେ ପକାଇଦେବାକୁ ଚାହୁଁଛୁ ଯେ, ଦୟାକରି ଆମେ ଯାହା ଆଲୋଚନା କଲେ, ତାହା ବାହାରେ କାହାକୁ ପ୍ରକାଶ କରିବେ ନାହିଁ |

Any questions?
ଆଉ କିଛି ପ୍ରଶ୍ନ ଅଛି କି ?

Thank you for your time.
ଆପଣ ସମୟ ଦେଇଥିବାରୁ ବହୁତ ବହୁତ ଧନ୍ୟବାଦ |
