# Supplementary material for: The RANI Project: A socio-normative intervention to reduce anemia in Odisha, India: A formative research protocol
Source: Gates Open Res. 2018 May 10;2:15. Originally published 2018 Mar 20. [Version 2] doi: 10.12688/gatesopenres.12808.2 (PMC5906750; doi:10.12688/gatesopenres.12808.2)
Supplement: Supplementary file 1 [file gatesopenres-2-13894-s0000.tgz › fc760b8c-8c57-4732-9bf6-83edefcf24a1.zip!/Supplementary file 2.pdf]

Key Informant Interview Guide/ପ୍ରମୁଖ ସୂଚନାଦାତାଙ୍କୁ ସହିତ ସାକ୍ଷାତକାର ନିର୍ଦ୍ଦେଶାବଳୀ

Before Beginning the Interview:
ସାକ୍ଷାତକାର ଆରମ୍ଭ ପୂର୍ବରୁ:

- Welcome participant and introduce yourself
  ଅଂଶଗ୍ରହଣକାରୀଙ୍କୁ ସ୍ଵାଗତ କରନ୍ତୁ ଏବଂ ନିଜର ପରିଚୟ ଦିଅନ୍ତୁ |

Hello, and thank you for speaking with me today. My name is ______ and I am a field researcher at DCOR. I am conducting a research study to understand the attitudes and beliefs of people in this community.
ନମସ୍କାର, ଏବଂ ଆପଣ ଆଜି ମୋ ସହିତ କଥା ହେଉଥିବାରୁ ଆପଣଙ୍କୁ ଧନ୍ୟବାଦ ଦେଉଛି | ମୋ ନାମ ହେଉଛି ____________ଏବଂ ମୁଁ DCOR ସଂସ୍ଥାର ଜଣେ କ୍ଷେତ୍ର ଅନୁସନ୍ଧାନକାରୀ ଭାବେ କାମ କରୁଅଛି | ମୁଁ ଏହି ଗ୍ରାମରେ ଲୋକମାନଙ୍କର ମନୋଭାବ ଏବଂ ଧାରଣା ଗୁଡିକୁ ଜାଣିବା ପାଇଁ ଗୋଟିଏ ଅନୁସନ୍ଧାନ କରୁଅଛି |

Thank you so much for agreeing to participate and taking time out of your day.
ଆପଣମାନେ ଏହି ଆଲୋଚନାରେ ଭାଗ ନେବା ପାଇଁ ସହମତି ଦେଇଥିବାରୁ ଏବଂ ଆପଣ ଆଜି ସମୟ ବାହାର କରି ଆସିଥିବାରୁ ଧନ୍ୟବାଦ ଦେଉଛି |

Do you have any questions about the research and your participation before we start?
ଆମେ ଆଲୋଚନାଟି ଆରମ୍ଭ କରିବା ପୂର୍ବରୁ, ଏହି ଅନୁସନ୍ଧାନ ବିଷୟରେ ଏବଂ ଆପଣଙ୍କ ଅଂଶଗ୍ରହଣ କରିବା ଉପରେ କିଛି ପ୍ରଶ୍ନ ଅଛି କି ?

- Review key points, ethics and confidentiality policy:
  ମୁଖ୍ୟ ବିନ୍ଦୁଗୁଡିକ ଉପରେ, ଆଚାର ବିଚାର ଉପରେ ଏବଂ ଗୋପନୀୟତା ସମ୍ବନ୍ଧୀୟ ନୀତି ଗୁଡିକ ଉପରେ ଅବଲୋକନ କରନ୍ତୁ |

- You will not gain any direct benefit from participating in this research; however, we hope our results will be used to reduce anemia in Odisha.
  ଏହି ଅନୁସନ୍ଧାନରେ ଭାଗ ନେବା ଦ୍ବାରା ଆପଣଙ୍କୁ ସିଧା ସଳଖ ଭାବେ କୌଣସି ଲାଭ ମିଳିବ ନାହିଁ : କିନ୍ତୁ ଆମେ ଆଶା କରୁଛୁ, ଏହି ଅନୁସନ୍ଧାନର ଫଳାଫଳ ଓଡିଶାରେ ରକ୍ତହିନତା ଦୂର କରିବା ପାଇଁ ବ୍ୟବହାର କରାଯିବ |

- We do not anticipate you will experience any risks but please feel free to not answer any questions that you are uncomfortable with or to stop the interview at anytime.
  ଆମେ ଆଶା କରୁନାହୁଁ କି ଆପଣ ଏହି ଆଲୋଚନାରେ କିଛି ସଙ୍କଟ / ଅସୁବିଧା ଅନୁଭବ କରିବେ | ମାତ୍ର, ଯଦି କୌଣସି ପ୍ରଶ୍ନର ଉତ୍ତର ଦେବା ପାଇଁ ଆପଣ ଅସୁବିଧା ମନେ କରନ୍ତି, ତାହାହେଲେ ତାହାର ଉତ୍ତର ଦେବା ପାଇଁ ଆପଣ ବାଧ୍ୟ ନୁହଁନ୍ତି ବା ଯେକୌଣସି ସମୟରେ ଏହି ସାକ୍ଷାତକାରରୁ ଓହରି ଯାଇପାରିବେ |

- The discussion in this interview is completely confidential
  ସାକ୍ଷାତକାରରେ ଯାହା ବି କଥା ହେବା ତାହାକୁ ସମ୍ପୁର୍ଣ୍ଣ ରୁପେ ଗୋପନୀୟ ରଖାଯିବ |

- Your responses will be kept confidential and your name will not be cited on any written materials coming out of this study.
  ଆପଣଙ୍କ ଉତ୍ତରଗୁଡିକୁ ଗୋପନୀୟ ରଖାଯିବ ଏବଂ ଆପଣଙ୍କର ନାମ ଏହି ଅନୁସନ୍ଧାନ ଦ୍ଵାରା ପ୍ରସ୍ତୁତ କରାଯାଇଥିବା କୌଣସି ଲିଖିତ କାଗଜପତ୍ରରେ ଉଲ୍ଲେଖ କରାଯିବ ନାହିଁ |

Key Informant Interview Guide/ପ୍ରମୁଖ ସୂଚନାଦାତାଙ୍କୁ ସହିତ ସାକ୍ଷାତକାର ନିର୍ଦ୍ଦେଶାବଳୀ

- There are no rights or wrong answers to the questions I am going to ask you. All experiences are important
  ମୁଁ ପଚାରୁଥିବା ପ୍ରଶ୍ନର କୌଣସି ଠିକ୍ କିମ୍ବା ଭୁଲ୍ ଉତ୍ତର ନାହିଁ | ଆପଣଙ୍କ ସମସ୍ତ ଉତ୍ତର/ଅନୁଭବ ଗୁଡିକ ଗୁରୁତ୍ୱପୂର୍ଣ ଅଟେ |

- The interview is being tape recorded so that we can accurately capture what you're saying, but no one besides the research team will have access to it.
  ଏହି ସାକ୍ଷାତକାରଟି ଆମେ ରେକର୍ଡିଂ କରୁଛୁ ଯାହା ଫଳରେ ଆପଣ କହିଥିବା ସମସ୍ତ କଥାକୁ ଆମେ ଲେଖିପରିବୁ | କିନ୍ତୁ ଏହି ଅନୁସନ୍ଧାନ ସହିତ ଜଡିତ ନଥିବା ବ୍ୟକ୍ତି ଏହାକୁ ବ୍ୟବହାର କରିପାରିବେ ନାହିଁ |

- You are free not to answer any questions that make you feel uncomfortable or to stop the interview at any time
  କୌଣସି ପ୍ରଶ୍ନର ଉତ୍ତର ଦେବା ପାଇଁ ଆପଣ ଅସୁବିଧା ମନେ କରନ୍ତି, ତାହାର ଉତ୍ତର ଦେବା ପାଇଁ ଆପଣ ବାଧ୍ୟ ନୁହଁନ୍ତି ବା ଯେକୌଣସି ସମୟରେ ଏହି ସାକ୍ଷାତକାରଟିରୁ ଓହରି ଯାଇପାରିବେ |

- We expect the interview to last about one hour
  ଆମେ ଆଶା କରୁଛୁ ଏହି ସାକ୍ଷାତକାରଟି ଦେଢ଼ ଘଣ୍ଟା ପର୍ଯ୍ୟନ୍ତ ସମୟ ଲାଗିବ |

Warm-up Questions / ପ୍ରାରମ୍ଭିକ ପ୍ରଶ୍ନ

I'm new to this community, [name District], can you tell me a little bit about it? For example, what kind of food do people eat here? And what do people do to earn money?
ମୁଁ ଆପଣଙ୍କ ସମୁଦାୟରେ/ଗ୍ରାମରେ ନୂଆ, (ଜିଲ୍ଲାର ନାମ), ଆପଣଙ୍କ ସମୁଦାୟ/ଗ୍ରାମ ବିଷୟରେ କିଛି କହିପାରିବେ କି ? ଉଦାହରଣସ୍ୱରୂପ, କେଉଁ ପ୍ରକାରର ଖାଦ୍ୟ ଏଠାରେ ଲୋକମାନେ ଖାଆନ୍ତି ? ଏବଂ ଲୋକମାନେ ରୋଜଗାର ପାଇଁ କଣସବୁ କରନ୍ତି ?

Women's Role in the Community/ଗ୍ରାମରେ/ସମୁଦାୟରେ ମହିଳାମାନଙ୍କର ଭୂମିକା

What are some of the typical things that women in this community do throughout the day? (probe: things like cooking, cleaning, taking care of kids, working outside the home, spending time with her husband).
ଆପଣଙ୍କ ଗ୍ରାମରେ/ସମୁଦାୟରେ ମହିଳାମାନେ ସାଧାରଣତଃ ଗୋଟିଏ ଦିନ ଭିତରେ କଣ ସବୁ କାର୍ଯ୍ୟ କରିଥାନ୍ତି ? (ପଚାରନ୍ତୁ: ଯେପରିକି ରୋଷେଇ କରିବା, ସଫା କରିବା, ପିଲାଙ୍କର ଯତ୍ନ ନେବା, ଘର ବାହାରେ କାର୍ଯ୍ୟ କରିବା, ସ୍ୱାମୀଙ୍କ ସହିତ ସମୟ ବିତାଇବା)

Do women in this community have enough energy or time to these things each day?
ପ୍ରତ୍ୟକ ଦିନ ଏହି କାର୍ଯ୍ୟ ସବୁ କରିବା ପାଇଁ, ଆପଣଙ୍କ ସମୁଦାୟରେ ମହିଳାମାନଙ୍କର ପର୍ଯ୍ୟାପ୍ତ ଶକ୍ତି ବା ସମୟ ଥାଏ କି ?

- Look after her kids?
  ଛୁଆମାନଙ୍କର ଯତ୍ନ ନେବା ?
- Work outside the home?
  ଘର ବାହାରେ କାର୍ଯ୍ୟ କରିବା ?
- Cook?
  ରୋଷେଇ କରିବା ?
- Spend time with her husband?
  ସ୍ୱାମୀଙ୍କ ସହିତ ସମୟ ବିତାଇବା ?

- Have more intimate time with her husband?
  ସ୍ୱାମୀଙ୍କ ସହିତ ଅଧିକ ଅନ୍ତରଙ୍ଗ ସମୟ ବିତାଇବା ?

How are men and women different in this community?
ଆପଣଙ୍କ ସମୁଦାୟ/ଗ୍ରାମରେ ପୁରୁଷ ଏବଂ ମହିଳାମାନେ ପରସ୍ପର ଠାରୁ କିପରି ଭିନ୍ନ ?

For women's life in general, are things getting better or worse?
ସାଧାରଣତଃ, ମହିଳାମାନଙ୍କର ଜୀବନରେ ଘଟୁଥିବା ଜିନିଷ ଗୁଡିକ ଭଲ ବା ଖରାପ ହେଉଛି କି ?

- in her role as a mother?
  'ମା' ହିସାବରେ ତାଙ୍କ ଭୂମିକା ?
- in her role as a wife?
  'ସ୍ତ୍ରୀ' ହିସାବରେ ତାଙ୍କ ଭୂମିକା ?
- in her role as a daughter in law?
  'ବୋହୂ' ହିସାବରେ ତାଙ୍କ ଭୂମିକା ?

What are some of the concerns that women in this community have? (probe: concerns about health, money, their family, themselves, their future)
ଆପଣଙ୍କ ସମୁଦାୟର ମହିଳାମାନଙ୍କର କଣ ସବୁ ଚିନ୍ତା ରହିଛି ? (ପଚାରନ୍ତୁ: ଯେପରିକି ସ୍ୱାସ୍ଥ୍ୟ, ଟଙ୍କା, ତାଙ୍କ ପରିବାର, ନିଜ ପାଇଁ, ଭବିଷ୍ୟତ ପାଇଁ ଚିନ୍ତା ସବୁ)

- Some people think that (woman's name) should first take care of her husband and kids before she worries about her own health. Other people think that she should first take care of her own health.
  କିଛି ଲୋକ ଭାବନ୍ତି କି, ଯେ (ମହିଳା ଙ୍କ ନାମ) ନିଜର ସ୍ୱାସ୍ଥ୍ୟଯତ୍ନ ପାଇଁ ଚିନ୍ତା କରିବା ପୁର୍ବରୁ ପ୍ରଥମେ ନିଜ ସ୍ୱାମୀ ଏବଂ ଛୁଆଙ୍କ ଯତ୍ନ ନେବା ଜରୁରୀ | ଅନ୍ୟ କିଛି ଲୋକ ଭାବନ୍ତି କି ଯେ ସେ ପ୍ରଥମେ ନିଜ ସ୍ୱାସ୍ଥ୍ୟର ଯତ୍ନ ନେବା ଜରୁରୀ |

How do you think most women in this community feel about that? What about most men?
ଆପଣଙ୍କ ଗ୍ରାମରେ ମହିଳା ମାନେ ଏହି ବିଷୟରେ କଣ ଭାବନ୍ତି ? ପୁରୁଷ ମାନେ କଣ ଭାବନ୍ତି ?

In some families in Odisha, women eat after their husband, children and mother in law eat. In others, women eat at the same time. What do you think about that? Is this changing?
ଓଡ଼ିଶାରେ ଥିବା କିଛି ପରିବାରରେ, ମହିଳାମାନେ ତାଙ୍କ ସ୍ୱାମୀ, ଛୁଆ ଏବଂ ଶାଶୂ ଖାଇସାରିଲା ପରେ ଖାଆନ୍ତି | ଅନ୍ୟ କିଛି ପରିବାରରେ, ମହିଳାମାନେ ସେମାନଙ୍କ ସହିତ ଏକା ସମୟରେ ଖାଇଥାଆନ୍ତି | ଏହି ବିଷୟରେ ଆପଣମାନେ କଣ ଭାବନ୍ତି ? ଏହା ପରିବର୍ତ୍ତନ ହେଉଛି କି?

Anemia in this Community/ ଆପଣଙ୍କ ସମୁଦାୟରେ / ରକ୍ତହୀନତା

Have you heard of anemia? (describe anemia briefly and show IFA tablets) Do lots of people have Anemia in your community? ଆପଣ ଆନେମିୟା ବିଷୟରେ ଶୁଣିଛନ୍ତି କି ? (ସଂକ୍ଷେପରେ ଆନେମିୟା କଣ ବର୍ଣ୍ଣନା କରନ୍ତୁ ଏବଂ ଆଇରନ ବଟିକା ଦେଖାନ୍ତୁ) | ଆପଣଙ୍କ ସମୁଦାୟରେ ବହୁଜଣଙ୍କର ରକ୍ତହୀନତା ଅଛି କି ?

Key Informant Interview Guide/ପ୍ରମୁଖ ସୂଚନାଦାତାଙ୍କୁ ସହିତ ସାକ୍ଷାତକାର ନିର୍ଦ୍ଦେଶାବଳୀ

How does the diet in your community contribute to anemia? (probe: for example do people here eat iron rich foods like meat or green leafy vegetables?)/ ଆପଣ ସମୁଦାୟରେ ଲୋକମାନଙ୍କର ଆହାର ରକ୍ତହୀନତା ହେବାରେ କିପରି ଯୋଗଦାନ କରିଥାଏ ? ପଚାରନ୍ତୁ: ଉଦାହରଣ ସ୍ବରୁପ ଏଠାରେ ଲୋକମାନେ ଆଇରନଯୁକ୍ତ ଖାଦ୍ୟ ଯେପରି ମାଂସ କିମ୍ବା ସବୁଜ ପନିପରିବା ଖାଆନ୍ତି କି ?

Knowledge about Anemia/ରକ୍ତହୀନତା ବିଷୟରେ ଜ୍ଞାନ

Do people in your community know that lot of women have anemia? How much do they know about it? Probe : health risk like fatigue, risk for the baby ବହୁତଜଣ ମହିଳାମାନଙ୍କର ରକ୍ତହୀନତା ଅଛି ବୋଲି ଆପଣଙ୍କ ସମୁଦାୟ/ଗ୍ରାମରେ ଲୋକମାନେ ଜାଣନ୍ତି କି ? | ସେମାନେ ଏହା ବିଷୟରେ କେତେ ଜାଣିଛନ୍ତି ? (ପଚାରନ୍ତୁ: ସ୍ୱାସ୍ଥ୍ୟ ସଙ୍କଟ ଯେପରିକି କ୍ଲାନ୍ତ ଅନୁଭବ କରିବା, ପେଟରେ ଥିବା ଛୁଆ ପ୍ରତି ବିପଦ)

IFA Availability/ଆଇରନ ବଟିକାର ଉପଲବ୍ଧତା

Can you tell me who distributes the tablets and how? (probe: Are they private or government distributors?/ଆପଣ ମୋତେ କହି ପରିବେ କି ଆଇରନ ବଟିକା କିଏ ଏବଂ କିପରି ବଣ୍ଟନ କରନ୍ତି ?(ପଚାରନ୍ତୁ: ସେମାନେ ସରକାରୀ ନା ବେସରକାରୀ ବଣ୍ଟନକାରୀ)

Typically, how much do IFA tablets cost? Is this cost a lot to people in the community? How many tablets does a person get at a time/ସାଧାରଣ ଭାବେ ଆଇରନ ବଟିକାର ମୂଲ୍ୟ କେତେ ? ଏହାର ମୂଲ୍ୟ ଆପଣଙ୍କ ସମୁଦାୟର ଲୋକଙ୍କ ପାଇଁ ବହୁତ ଅଧିକ କି ? ଜଣେ ବ୍ୟକ୍ତି ଏକ ସମୟରେ କେତୋଟି ବଟିକା ପାଆନ୍ତି ?

Barriers and Facilitators to IFA use/ଆଇରନ ବଟିକାର ବ୍ୟବହାରକୁ ନେଇ ପ୍ରତିବନ୍ଧକ ଏବଂ ସୁବିଧା ସବୁ

What do people in the community know about the benefits of taking IFA tablets? (probe: do most people in this community know about the connection between IFA tablets and anemia?)/ଆଇରନ ବଟିକାର ନିୟମିତ ଖାଇବାର ଭଲ ପ୍ରଭାବଗୁଡିକ ଉପରେ ଗ୍ରାମର ଲୋକମାନେ କଣ ଜାଣିଛନ୍ତି ? (ପଚାରନ୍ତୁ: ଆଇରନ ବଟିକା ଏବଂ ରକ୍ତହୀନତା ମଧ୍ୟରେ ଥିବା ସମ୍ବନ୍ଧକୁ ଗ୍ରାମରେ ଥିବା ଅଧିକାଂଶ ଲୋକ ଜାଣନ୍ତି କି ?

What do people think are the drawbacks of taking IFA tablets? (probe: what are some of the potential side effects of the tablets? How do they feel about these side effects?)/ଆଇରନ ବଟିକା ଖାଇବାକୁ ନେଇ କଣ ସବୁ ଖରାପ ପ୍ରଭାବ ହୋଇପାରେ ବୋଲି ଲୋକମାନେ ଭାବନ୍ତି ? (ପଚାରନ୍ତୁ: ଆଇରନ ବଟିକା ଖାଇବା ଦ୍ୱାରା କଣ ସବୁ ପାର୍ଶ୍ୱ ପ୍ରତିକ୍ରିୟା ପଡେ? ଏହି ପାର୍ଶ୍ୱ ପ୍ରତିକ୍ରିୟାକୁ ନେଇ ଲୋକମାନେ କଣ ସବୁ ଭାବିଥାନ୍ତି ?)

IFA Norms/ଆଇରନ ବଟିକା ଖାଇବାର ନିୟମାବଳୀ

Who is typically expected to take IFA tablets? (probe: Adolescent , all women, only pregnant women? କିଏ ମୁଖ୍ୟତଃ ଆଇରନ ବଟିକା ଖାଇବା କଥା ?(ପଚାରନ୍ତୁ: କିଶୋରୀ ବାଳିକା, ସବୁ ମହିଳା, କେବଳ ଗର୍ଭବତୀ ?)

Key Informant Interview Guide/ପ୍ରମୁଖ ସୂଚନାଦାତାଙ୍କୁ ସହିତ ସାକ୍ଷାତକାର ନିର୍ଦ୍ଦେଶାବଳୀ

Please think about most women who live in this community. How many of them take IFA tablets? Some? Few? Most? ଏହି ଗ୍ରାମରେ ରହୁଥିବା ଅଧିକାଂଶ ମହିଳାଙ୍କ ବିଷୟରେ ଭାବନ୍ତୁ | ସେମାନଙ୍କ ମଧ୍ଯରୁ କେତେଜଣ ଆଇରନ ବଟିକା ଖାଆନ୍ତି? କିଛି ଜଣ ? ଅଳ୍ପ ଜଣ ? ଅଧିକାଂଶ ଜଣ ?

Please think about most Adolescent who live in this community. How many of them take IFA tablets? Some? Few? Most? ଏହି ଗ୍ରାମରେ ରହୁଥିବା ଅଧିକାଂଶ କିଶୋରୀ ବାଳିକାଙ୍କ ବିଷୟରେ ଭାବନ୍ତୁ | ସେମାନଙ୍କ ମଧ୍ଯରୁ କେତେଜଣ ଆଇରନ ବଟିକା ଖାଆନ୍ତି ? କିଛି ଜଣ ? ଅଳ୍ପ ଜଣ ? ଅଧିକାଂଶ ଜଣ ?

Role of Aganwadi worker, ANM and ASHA/ଅଙ୍ଗନବାଡି କର୍ମୀ, ଏ.ଏନ୍.ମ୍. ଓ ଆଶାର ଭୂମିକା

Can you tell us a little bit about the Aganwadi workers, the ANMs and the ASHA? What is their relationship with each other/ଆପଣ ଆମକୁ ଅଙ୍ଗନବାଡି, ଏ.ଏନ୍.ମ୍. ଏବଂ ଆଶା ବିଷୟରେ କିଛି କହିପାରିବେ କି ? ତାଙ୍କର ପରସ୍ପର ସହିତ କଣସବୁ ସମ୍ପର୍କ ଅଛି ?
What is their relationship with the self-help groups/ତାଙ୍କର ସ୍ୱୟଂ ସହାୟକ ଗୋଷ୍ଠି ସହିତ କଣସବୁ ସମ୍ପର୍କ ଅଛି ?

Self Help Groups/ସ୍ୱୟଂ ସହାୟକ ଗୋଷ୍ଠି

Are there any SHGs in this village? What kinds of things do they discuss? What type of women join? Why do they join?)/ଗ୍ରାମରେ କୌଣସି ସ୍ୱୟଂ ସହାୟକ ଦଳସବୁ ଅଛି କି ? ସେମାନେ କେଉଁ ପ୍ରକାରର ଜିନିଷ ଆଲୋଚନା କରନ୍ତି | କେଉଁ ପ୍ରକାରର ମହିଳା ଏଥିରେ ଯୋଗ ଦିଅନ୍ତି ? କାହିଁକି ଏଥିରେ ଯୋଗ ଦିଅନ୍ତି ?)

What are the attitudes within the self-help groups? (probe: do people view it as helpful? In what ways do you think it is helpful?)/ସ୍ୱୟଂ ସହାୟକ ଗୋଷ୍ଠି ଭିତରେ ସଦସ୍ୟ ମାନଙ୍କର କେଉଁପ୍ରକାର ମନୋଭାବ ଥାଏ ? (ପଚାରନ୍ତୁ: ଲୋକମାନେ ସ୍ୱୟଂ ସହାୟକ ଗୋଷ୍ଠିକୁ ସାହାଯ୍ୟକାରୀ ବୋଲି ଦେଖନ୍ତି କି ? ଆପଣ କଣ ଭାବୁଛନ୍ତି ଏହା କେମିତି ଭାବରେ ସାହାଯ୍ୟକାରୀ?)

Health Care System/ ସ୍ୱାସ୍ଥ୍ୟ ସେବା ବ୍ୟବସ୍ଥା

Where can someone get information about health? (Probe: health clinic, from family members)/ସ୍ୱାସ୍ଥ୍ୟ ସମ୍ବନ୍ଧୀୟ ସୂଚନାସବୁ କେଉଁଠାରୁ ପାଇପାରିବେ ? (ପଚାରନ୍ତୁ: ସ୍ୱାସ୍ଥ୍ୟ କେନ୍ଦ୍ର, ପରିବାର ସଦସ୍ୟ) Are these the same places where women can find information about anemia or IFA tablets? ଏଗୁଡିକ ସେଇସବୁ ଯାଗା ଯେଉଁଠାରୁ ମହିଳାମାନେ ରକ୍ତହୀନତା ଏବଂ ଆଇରନ ବଟିକା ବିଷୟରେ ସୂଚନା ପାଇପାରିବେ ?

Are traditional healers or doctors more commonly used? (Probe: why is one is better than the other?)/ ଲୋକମାନେ ସାଧାରଣତଃ ବୈଦ୍ୟ ନା ଡାକ୍ତରଙ୍କ ପାଖକୁ ଚିକିତ୍ସା ପାଇଁ ଯାଆନ୍ତି ?(ପଚାରନ୍ତୁ: କାହିଁକି ଜଣେ ଅନ୍ୟ ଜଣକ ଠାରୁ ଭଲ ?)

Key Informant Interview Guide/ପ୍ରମୁଖ ସୂଚନାଦାତାଙ୍କୁ ସହିତ ସାକ୍ଷାତକାର ନିର୍ଦ୍ଦେଶାବଳୀ

Are private or government run health clinics more commonly used? (Probe: why is one is better than the other? How about to get screened for anemia? How about to get IFA tablets?)/ ଲୋକମାନେ ସାଧାରଣତଃ ବେସରକାରୀ ନା ସରକାରୀ ସ୍ୱାସ୍ଥ୍ୟକେନ୍ଦ୍ରକୁ ଚିକିତ୍ସା ପାଇଁ ଯାଆନ୍ତି ? (ପଚାରନ୍ତୁ: କାହିଁକି ଗୋଟିଏ ଅନ୍ୟ ଠାରୁ ଭଲ ? ରକ୍ତହୀନତା ପାଇଁ କେମିତି ଯାଞ୍ଚ କରାଇବେ? କେମିତି ଆଇରନ ବଟିକା ପାଇପାରିବେ ?)

Prior Interventions/ ପୂର୍ବରୁ ଚାଲିଥିବା କାର୍ଯ୍ୟକ୍ରମ

Have any programs in this community tried to reduce anemia or increase IFA tablet use? Can you describe these programs to me? (probe: How well liked were they? What worked well for the programs what didn't)/ରକ୍ତହୀନତା କମାଇବା ବା ଆଇରନ ବଟିକାର ବ୍ୟବହାରକୁ ସୁଧାରିବା ପାଇଁ ଏହି ସମୁଦାୟରେ କିଛି କାର୍ଯ୍ୟକ୍ରମ ଚାଲୁଅଛି କି ? ଏହି କାର୍ଯ୍ୟକ୍ରମ ଗୁଡିକ ବିଷୟରେ ମତେ ବର୍ଣ୍ଣନା କରିପାରିବେ କି ? (ପଚାରନ୍ତୁ: ଏହି କାର୍ଯ୍ୟକ୍ରମଗୁଡ଼ିକ ଲୋକମାନଙ୍କ ଦ୍ୱାରା କେତେ ଭଲଭାବରେ ଗ୍ରହଣ ଯୋଗ୍ୟ ହୋଇଥିଲା ? କଣସବୁ ଏହି କାର୍ଯ୍ୟକ୍ରମ ପାଇଁ ଭଲ କାମ କରିଥିଲା ଏବଂ କଣ ଭଲ କାମ କରିନଥିଲା ?

Intervention Feasibility/କାର୍ଯ୍ୟକ୍ରମର ବ୍ୟବହାର୍ଯ୍ୟତା

What do you think is good way to reduce anemia in your community? ଆପଣଙ୍କ ସମୁଦାୟରେ ରକ୍ତହୀନତା ଦୂର କରିବା ପାଇଁ, ଆପଣ କଣ ଉତ୍ତମ ପଦ୍ଧତି ବୋଲି ଭାବୁଛନ୍ତି |

Closing/ସମାପନ

That is the end of the questions I have for you, but do you have anything else you'd like to add to the discussion? Any little stories about anemia or IFA use in this community?
ମୋର ପ୍ରଶ୍ନ ପଚାରିବା ଶେଷ ହେଲା, କିନ୍ତୁ ଆପଣମାନେ ଏହି ଆଲୋଚନାରେ ଆଉ କିଛି କହିବାକୁ ଚାହୁଁଛନ୍ତି କି ? ରକ୍ତହୀନତା କିମ୍ବା ଆଇରନ ବଟିକା ଉପରେ, ଆପଣଙ୍କ ଗ୍ରାମରେ ହୋଇଥିବା କିଛି ଘଟଣା ବା କାହାଣୀ କହିବେ କି ?

As a reminder, please do not share anything we spoke about today with anyone outside of this group.
ଆପଣଙ୍କୁ ମନେ ପକାଇଦେବାକୁ ଚାହୁଁଛୁ ଯେ, ଦୟାକରି ଆମେ ଯାହା ଆଲୋଚନା କଲେ, ତାହା ବାହାରେ କାହାକୁ ପ୍ରକାଶ କରିବେ ନାହିଁ |

Any questions?
ଆଉ କିଛି ପ୍ରଶ୍ନ ଅଛି କି ?

Thank you for your time.
ଆପଣ ସମୟ ଦେଇଥିବାରୁ ବହୁତ ବହୁତ ଧନ୍ୟବାଦ |
