# Supplementary material for: The RANI Project: A socio-normative intervention to reduce anemia in Odisha, India: A formative research protocol
Source: Gates Open Res. 2018 May 10;2:15. Originally published 2018 Mar 20. [Version 2] doi: 10.12688/gatesopenres.12808.2 (PMC5906750; doi:10.12688/gatesopenres.12808.2)
Supplement: Supplementary file 1 [file gatesopenres-2-13894-s0000.tgz › fc760b8c-8c57-4732-9bf6-83edefcf24a1.zip!/Supplementary file 3.pdf]

Structured Observation Check Lists – Formative Research in Odisha, India

ANC Clinics & Kiosks/IFA dispensaries:

- Ask to buy IFA tablets. What kinds do they have? What is the price? Do they ever run out? Ask what they tell people when they give them the tablets (examples: dose, when to take, why they need to take them)
  ଆଇରନ ବଟିକା କିଣିବେ ବୋଲି କୁହନ୍ତୁ | କେଉଁ ପ୍ରକାରର ଆଇରନ ବଟିକା ସେମାନଙ୍କ ପାଖରେ ଅଛି ପଚାରନ୍ତୁ ? କେତେ ପଇସା ? ସେମାନଙ୍କର କେବେବି ଆଇରନ ବଟିକାର ଷ୍ଟକ ସରିଛି କି ? ପଚାରନ୍ତୁ, ସେମାନେ ଆଇରନ ବଟିକା ଦେଲାବେଳେ, ଲୋକମାନଙ୍କୁ ସେମାନେ କଣ କୁହନ୍ତି (ଉଦାହରଣ: କେତେଥର ଖାଇବେ, କେତେବେଳେ ଖାଇବେ, କାହିଁକି ଆଇରନ ବଟିକା ଖାଇବା ଦରକାର) ?

- Is it a government run clinic or private?
  ଏହା ସରକାରୀ ନା ବେସରକାରୀ କ୍ଲିନିକ ?

- Are there educational materials about anemia or IFA tablets in the clinic? Any other health related materials? If so, take pictures of all anemia and IFA related materials and describe it
  ରକ୍ତହୀନତା କିମ୍ବା ଆଇରନ ବଟିକା ସମ୍ବନ୍ଧୀୟ ଶିକ୍ଷଣୀୟ ଜିନିଷ କ୍ଲିନିକରେ ଅଛି କି ? ଅନ୍ୟ କୌଣସି ସ୍ୱାସ୍ଥ୍ୟ ସମ୍ବନ୍ଧୀୟ ସାମଗ୍ରୀ ଅଛି କି ? ଯଦି ଅଛି, ରକ୍ତହୀନତା ଏବଂ ଆଇରନ ବଟିକା ସମ୍ବନ୍ଧୀୟ ଶିକ୍ଷଣୀୟ ଜିନିଷ ଗୁଡିକର ଫଟୋ ଉଠାନ୍ତୁ ଏବଂ ବର୍ଣ୍ଣନା କରନ୍ତୁ |

- Take pictures of the clinic itself and describe it
  କ୍ଲିନିକର ଫଟୋ ଉଠାନ୍ତୁ ଏବଂ ବର୍ଣ୍ଣନା କରନ୍ତୁ |

Self-help Groups:

- Where do the self-help groups meet? What is the composition of members (age/caste) ? How many participants? How often do they meet?
  କେଉଁଠାରେ ସ୍ୱୟଂ ସହାୟକ ଦଳର ସଭା ହୁଏ ? କେଉଁ ପ୍ରକାରର ସଦସ୍ୟ (ବୟସ / ଜାତି) ଦଳରେ ଅଛନ୍ତି ? କେତେଜଣ ସଦସ୍ୟ ଅଛନ୍ତି ? କେତେ ବ୍ୟବଧାନରେ ସେମାନେ ବୈଠକ କରନ୍ତି ?

- Describe what they discuss and do
  ସେମାନେ କଣ ଆଲୋଚନା ଏବଂ କାର୍ଯ୍ୟ କରନ୍ତି, ବର୍ଣ୍ଣନା କରନ୍ତୁ ?

- How well functioning does the group appear? What is the overall sentiment from participants and leaders? Are they happy with the group? Unhappy?
  ଦଳ କେତେ ଭଲ କାମ କରୁଛି ବୋଲି ଜଣା ପଡୁଛି ? ଅଂଶଗ୍ରହଣକାରୀ ଏବଂ ମୁଖ୍ୟଙ୍କ ମୋଟାମୋଟି ଭାବନା କଣ ? ସେମାନେ ଦଳସହିତ ଖୁସି ଅଛନ୍ତି କି ? ଖୁସି ନାହାଁନ୍ତି ?

Food markets & general community observation:

- Take photos of all food at the market
  ହାଟ / ବଜାରରେ ଥିବା ଖାଦ୍ୟ ପଦାର୍ଥ ଗୁଡିକର ଫଟୋ ଉଠାନ୍ତୁ |

- List out all of the food in a separate list
  ସବୁ ଖାଦ୍ୟପଦାର୍ଥର ଗୋଟିଏ ଅଲଗା ତାଲିକା ତିଆରି କରନ୍ତୁ ?

- Are there any health related materials in the area (posters, advertisements, brochures, etc.)? If so, take photos and describe it.
  ଏହି ଅଞ୍ଚଳରେ କିଛି ସ୍ୱାସ୍ଥ୍ୟ ସମ୍ବନ୍ଧୀୟ ଜିନିଷ ଗୁଡିକ ଅଛି କି (ପୋଷ୍ଟର, ବିଜ୍ଞାପନ, ପତ୍ରିକା, ଇତ୍ୟାଦି) ? ଯଦି ଅଛି, ତାହାର ଫଟୋ ଉଠାନ୍ତୁ ଏବଂ ବର୍ଣ୍ଣନା କରନ୍ତୁ ?
