# Supplementary material for: The RANI Project: A socio-normative intervention to reduce anemia in Odisha, India: A formative research protocol
Source: Gates Open Res. 2018 May 10;2:15. Originally published 2018 Mar 20. [Version 2] doi: 10.12688/gatesopenres.12808.2 (PMC5906750; doi:10.12688/gatesopenres.12808.2)
Supplement: Peer review history

# Peer review: Referee response for version 1

**DOI:** 10.21956/gatesopenres.13875.r26323

**Published:** 2018-04-10

**Authors:** Ashraful Alam

**Affiliations:** 1 Sydney School of Public Health, University of Sydney, Sydney, NSW, Australia

The paper presents a plan of a formative research aiming to generate contextual evidence for designing and implementing a feasible and efficient intervention to reduce iron-deficiency anemia among women of reproductive age and pregnant women in Odisha, India. The authors will use qualitative methods (interviews, discussions, observation and visual tool) to collect the data. The paper addresses an important health issue particularly relevant to low-income settings and the populations with certain dietary perceptions and practices hindering iron-rich food intake, such as many Indian communities. At the research approach level, the study will benefit the public health researchers and program implementers by providing with conceptual and methodological insights into designing formative research to inform culturally appropriate community-based intervention to improve maternal nutrition. Overall, the protocol is well-designed that provides details of every step of the research. I have a few comments that the authors may consider to improve the research protocol.

  

1. Page 1. Abstract. The authors say that to improve adoption and sustainability of the intervention, they "will conduct policy dialogue with stakeholders throughout the formative research." Formative research will be conducted prior to the start of the intervention. How the dialogue during the formative research will improve the intervention adoption? The authors may like to elaborate on this.
2. Page 6. Observational Data. Observation provides very useful data on actual behaviors. At the same time, the method poses immense danger of data distortion caused by the presence of the observer and study populations' prior awareness of the observation. The authors should articulate a strategy of minimizing the bias, in particular how they will conceal the observation schedule from the clinic staff/clients, and what measures they will take to refrain the staff from improved performance to 'satisfy' the observers (i.e. communications about anemia and/or IFA supplementation).
3. Pg 8. Site selection. Please provide the reason for selecting the formative research villages from outside the panned RCT villages. In my view, the RCT villages would provide more relevant data to inform the RCT design.
4. There are too many passive sentences in the paper that make it difficult to understand about the actor. Please turn as many passive sentences as possible into active sentences so the readers understand who will do what.

I have read this submission. I believe that I have an appropriate level of expertise to confirm that it is of an acceptable scientific standard.

# Peer review: Referee response for version 1

**DOI:** 10.21956/gatesopenres.13875.r26325

**Published:** 2018-03-29

**Authors:** Nilesh Chatterjee, Genevie Fernandes

**Affiliations:** 1 Water and Sanitation Program, World Bank, New Delhi, Delhi, India; 2 Usher Institute of Population Health Sciences & Informatics, University of Edinburgh, Edinburgh, UK

**General comments:**

 Authors provide a detailed description and rationale of the qualitative research protocol. Article can be accepted with minor revisions or changes.

 The aim of this formative assessment study is to gather data and information that will help in the design, testing, and refinement of an intervention that can be feasibly and effectively implemented to reduce iron-deficiency anemia among women of reproductive age and pregnant women in Odisha, India.

 To achieve the above, the formative assessment aims to do the following:

1. At the community level, to identify key contextual, administrative, policy, and service environments that either facilitate or hinder access to and use of IFA supplements among women of reproductive age and pregnant women.
2. At the interpersonal and individual levels, to identify norms, referent groups, and the extent to which they act as barriers or facilitators to IFA use and compliance among reproductive age women and pregnant women
3. Finally, to design, pilot test, and subsequently modify and finalize an intervention to increase IFA use and compliance and to reduce anemia among reproductive age women (with a focus on transforming harmful norms and promoting beneficial norms).

In this formative assessment, the researchers will use various methods to gather qualitative data such as in-depth interviews, focus groups, Participatory Ethnographic Evaluation and Research (PEER) interviews, and observations in various venues. The authors propose to not only analyze the information emerging or coming in from various methods but also triangulate the information from these different methods to arrive at an understanding of the phenomenon. This can take some time.

 However, the authors state that the formative assessment will be conducted from March to December, 2018. And within this time duration not only will there be data collection using various methods but also a convening workshop to be conducted in July, where, a first draft of the intervention plan is to be formulated; and pilot testing of various components of the intervention to determine its feasibility and effectiveness. The results of these pilot evaluations will inform and refine the final intervention, which is to be developed by December, 2018. While we find that the formative assessment is meticulously planned, we also request the authors to re-examine and re-visit the proposed time duration for all of these activities. Addressing anemia in women of reproductive age is critical. This is an important project and the authors/ research team have spent time in thinking through various components; made an elaborate plan to gather data and the formative phase must be done in the best possible manner even if that means increasing the time allocated.

**Specific comments** are provided below for each section of the article. The authors may use this to re-examine the article and make some minor revisions.

**Comments on the Introduction section:**

1. Please cite appropriate references for the below-mentioned sentences on page 3, column 1, paragraph 3, line 3.
  Reasons for these are multifactorial and include consumption of a predominantly plant based diet; micronutrient deficiencies, such as vitamin B12; and hookworm and malarial infections. Physical and cognitive productivity losses associated with anemia in India are estimated to be 6% of Gross Domestic Product (GDP).
2. It would be helpful if the authors stated the year in which the Ministry of Health and Family Welfare launched the National Iron+ Initiative Program and the Adolescent Girls Anemia Control Program (see page 3). Were there programs before this? Is it possible to provide readers a historical sense of government initiatives for addressing anemia.
3. Please cite appropriate reference for the below given sentence on page 3, column 2, paragraph 3.
  Data from the 2015–2016 cycle of the India Demographic Health Data show that 91 percent of pregnant mothers reported that they received IFA supplements during their last pregnancy but only 37 percent consumed them for more than 100 days during pregnancy.
4. Check the link for reference number 24 – Bentley and Parekh 1998. It appears to be unavailable. In the reference list, revise the spelling ‘Prakh’ to Parekh.
5. On page 3, column 2, last paragraph, please specify that the qualitative study was conducted in developing countries.

 

**Comments on Research methods and analysis:**

1. On page 4, please cite appropriate references for sentences under *research setting*.
2. On page 4, under research settings, it will be helpful to include data for percentage of women that received antenatal check-ups and institutional deliveries in the state, to get a sense of women’s access to government health services.
3. Authors have stated on Page 8 (lines 10 and 11) that a pilot study will be conducted in Bhubaneshwar, Could the authors describe this in more detail?
4. Also for the geographic locations of the formative assessment as well as the planned intervention; it will be helpful to have a map or some such graphic illustration of the state and the selected locations for the formative assessment.
5. Since the state of Odisha has a sizeable tribal population, it would be interesting to see how the researchers have incorporated inputs from tribal leaders or other key informants in this group.
6. In general, for all groups or villages, are local panchayati raj institution (PRI) members covered under government representatives?
7. The authors have included two different age groups of women and men for focus groups (younger and older). This is helpful in the formative phase.
8. We understand that observing private providers is important in the Indian context where a large portion of health care accessed by households is privatized? However, the authors could explain this process in slightly more detail in the paper.
9. Could the authors provide an approximate number of planned observations for each of the four venues? Also, it will be helpful to describe where these venues are? What is the catchment area?
10. Could the authors cite any study where the method of perceptual mapping has been used; this will provide the readers with an example of the process and efficacy of this research method.

 

**Comments on Evidence-informed policy dialogue:**

1. On page 8, add ‘we’ in the following sentence:
  To achieve this, **we** will build a policy dialogue platform based……

 

**Comments on Site selection:**

1. There is one concern about the selected site of Angul district. The selected district ‘Angul’ is stated to be three hours away from the capital city of Bhubaneshwar. Furthermore, as per findings from NFHS (2016) (hyperlinked report), almost 70% of pregnant women in this district had 4 ANC visits, 90% of women had institutional deliveries, and 92% of births have been conducted by a skilled birth attendant. Because of proximity to a major city and the NFHS findings, there is a concern this site may not be representative. We understand that representativeness may not always be a major criterion in qualitative research; however, the attributes of this district, the ideation, intention and behaviors of the people in this district may not match that of other districts in the interior of the state. Will the findings of this study then be applicable in the context of rural villages and districts or the entire state? However, despite a concern about access to better information and maybe health care services or providers, 44% of all women and 58% of pregnant women in Angul were reported as anemic in the same NFHS data; and only 38% of pregnant women reported consuming IFA tablets. It is possible that Angul was selected because it represents both sides of Odisha – the proximity to urban and urban-facing part as well as the rural-facing part. The authors may want to explain the site selection in a more nuanced manner.

 

**Comments on Data collection instruments:**

1. In the focus group guide, authors may want to include a few questions on aspirations of women and men. This will give an insight into possible motivations for behaviors around pregnancy and childbirth. There is a general trend towards an aspirational and more consumerism-driven lifestyle in many parts of India, especially in the smaller (rapidly growing) cities and towns.

We have read this submission. We believe that we have an appropriate level of expertise to confirm that it is of an acceptable scientific standard.
